# Osteomodulin downregulation is associated with osteoarthritis development

**DOI:** 10.1038/s41413-023-00286-5

**Published:** 2023-09-20

**Authors:** Jérémie Zappia, Qiao Tong, Renée Van der Cruyssen, Frederique M. F. Cornelis, Cécile Lambert, Tiago Pinto Coelho, Juliane Grisart, Erika Kague, Rik J. Lories, Marc Muller, Dirk Elewaut, Chrissy L. Hammond, Christelle Sanchez, Yves Henrotin

**Affiliations:** 1 MusculoSKeletal Innovative Research Lab, Center for Interdisciplinary Research on Medicines, Université de Liège, Liège, Belgium; 2 School of Physiology, Pharmacology, and Neuroscience, University of Bristol, Bristol, UK; 3 Department of Biomedical Molecular Biology, Ghent University, Ghent, Belgium; 4 Laboratory for Molecular Immunology and Inflammation, Department of Rheumatology, Ghent University Hospital, Ghent, Belgium; 5 Laboratory of Tissue Homeostasis and Disease, Skeletal Biology and Engineering Research Center, Department of Development and Regeneration, KU Leuven, Leuven, Belgium; 6 Cardiovascular Sciences, Groupe Interdisciplinaire de Génoprotéomique Appliquée, Université de Liège, Liège, Belgium; 7 Division of Nephrology, CHU of Liège, Université de Liège, Liège, Belgium; 8 Artialis SA, Tour GIGA, CHU Sart-Tilman, Liège, Belgium; 9 Division of Rheumatology, University Hospitals Leuven, Leuven, Belgium; 10 Laboratoire d’Organogenèse et Régénération, Groupe Interdisciplinaire de Génoprotéomique Appliquée, Université de Liège, Liège, Belgium; 11 Physical Therapy and Rehabilitation Department, Princess Paola Hospital, Vivalia, Marche-en-Famenne, Belgium

**Keywords:** Bone, Homeostasis

## Abstract

Abnormal subchondral bone remodeling leading to sclerosis is a main feature of osteoarthritis (OA), and osteomodulin (OMD), a proteoglycan involved in extracellular matrix mineralization, is associated with the sclerotic phenotype. However, the functions of OMD remain poorly understood, specifically in vivo. We used *Omd* knockout and overexpressing male mice and mutant zebrafish to study its roles in bone and cartilage metabolism and in the development of OA. The expression of *Omd* is deeply correlated with bone and cartilage microarchitectures affecting the bone volume and the onset of subchondral bone sclerosis and spontaneous cartilage lesions. Mechanistically, OMD binds to RANKL and inhibits osteoclastogenesis, thus controlling the balance of bone remodeling. In conclusion, OMD is a key factor in subchondral bone sclerosis associated with OA. It participates in bone and cartilage homeostasis by acting on the regulation of osteoclastogenesis. Targeting OMD may be a promising new and personalized approach for OA.

## Introduction

Osteoarthritis (OA) is a degenerative joint disease with a high prevalence that affected 527.8 million people worldwide in 2019.^1^ OA, as a major cause of disability, is a public health challenge and a rising societal burden due to the aging population and increasing life expectancy.^1^ OA is a heterogeneous disease originating from multifactorial causes with different subtypes of patients linked to distinct phenotypes.^2,3^ It is associated with pathologic changes in all joint tissues, including subchondral bone, cartilage, meniscus and synovium.^4^ One of the main OA features is subchondral bone sclerosis, which results from impaired subchondral bone remodeling driven by excessive mechanical loading. Bone sclerosis is associated with abnormalities in bone matrix biochemistry and mechanical properties that contribute to OA physiopathology. Among these abnormalities, loss of matrix elasticity, abnormal mineralization, modification of the proteomic landscape with impaired cytokine production such as increased transforming growth factor β and interleukin 6 levels, overexpression of proteases, and decreased synthesis of small proteoglycans are well documented.^5–8^ These changes are associated with the bone-driven OA phenotype.^3,4^

The small leucine-rich proteoglycans (SLRPs) are intricately related to the physical properties of bone and can be used as a fingerprint of its health status.^9^ The majority of SLRPs control the organization of collagen fibrils and, through the extracellular matrix (ECM), interact directly with cytokines, acting as a reservoir and a regulator of their bioavailability.^10–15^ Mice deficient for the SLRPs biglycan, fibromodulin, epiphycan, lumican, and chondroadherin demonstrated the protective role of these proteoglycans on the bone and cartilage matrix or osteoblasts; many of the knockout mutants showed premature or more pronounced OA.^16–24^ In contrast, knockout of opticin was associated with an inhibition of cartilage damage in an OA model.^25^ Until now, the role played by osteomodulin (OMD), also known as osteoadherin, in OA physiopathology has been poorly documented.

OMD is a keratan sulfate proteoglycan and a member of the SLRP family. OMD was originally isolated and characterized from bone and shown to be strongly expressed by osteoblasts.^26,27^ Although *OMD* is considered to be mainly expressed in bone, its expression has been observed in other cell types, such as articular chondrocytes and fibrochondrocytes.^28^ It is involved in the mineralization process by binding to osteoblasts through the α_V_β_3_ integrin and by stabilizing bone morphogenetic protein 2 (BMP2) ligands on their membrane receptors.^27,29,30^ A secretome analysis comparing osteoblasts from sclerotic and nonsclerotic areas of OA patients performed in our laboratory has shown that OMD is one of the major proteins downregulated by sclerotic osteoblasts in culture.^8^ Mature osteoblasts show enhanced expression of *OMD* when osteoclast activity is increased.^31^

For the first time, *Omd* knockout mice, referred to as KO mice, and mice with *Omd* gain-of-function in osteoblasts, hereafter referred to as UPs, were used to decipher the roles of *Omd* in bone remodeling and OA physiopathology. We followed the development of OA in aging mice and after destabilization of the medial meniscus (DMM). We focused on the subchondral bone, as a lack of OMD was reported to be related to bone sclerosis.^8^ In addition, we used the zebrafish model to study the role of *omd* in development and bone remodeling. Finally, using in vitro models, we deepened our investigation of the relationship between OMD and osteoclastogenesis.

## Results

### General growth characteristics of *Omd* KO and UP mice

*Omd* KO mice had a lower weight and body size than WT mice only at 4 months. UP mice had a smaller weight than the WT at 4 months, while their body size was not significantly different (Fig. S1A, B). At 8 and 16 months, weight and size were similar in all genotypes (Fig. S1A, B). The UP mice displayed a longer femur than the KO mice at 8 months. The femoral length evolved differently over time between the genotypes, with the UP mice reaching the mature size the soonest. Their femoral length was significantly increased at 8 months compared to the length at 4 months. At 16 months, each genotype reached a similar femoral length (Fig. S1C, D). Apart from some differences in their general growth characteristics over time, overall modifications of the level of *Omd* expression did not induce gross phenotypes.

### OMD is mainly localized in bone and calcified cartilage in mouse knee joint tissues

**Fig. 1:**
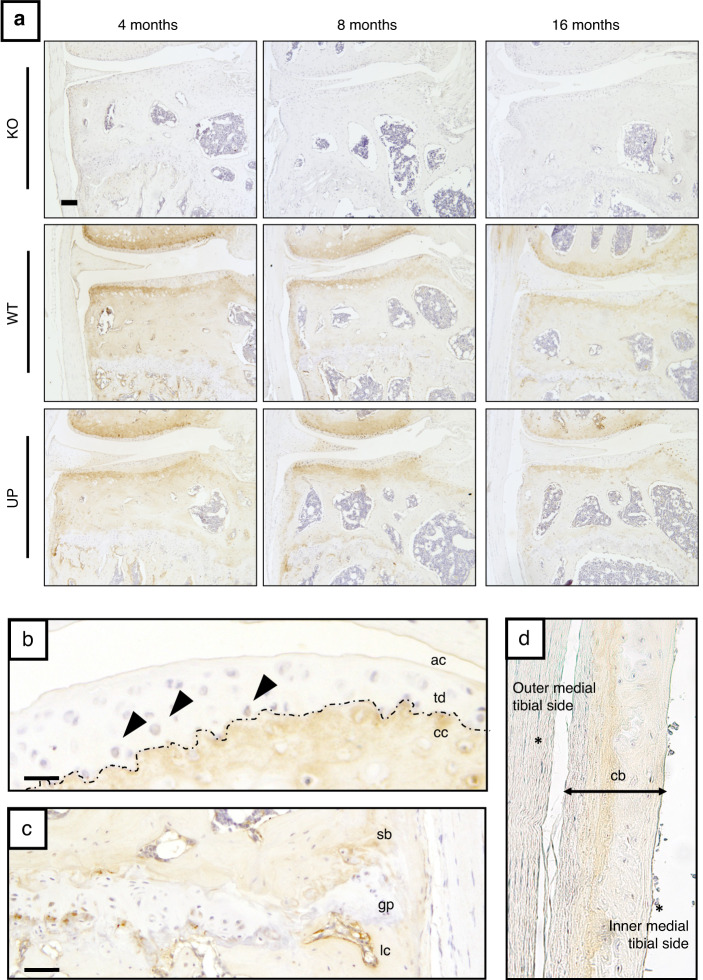
Localization of OMD in the murine knee joint. **a** Immunostaining of OMD (in brown) in the knee joint (medial tibial plateau) of KO, WT, and UP male mice at 4, 8, and 16 months. Scale bar = 100 µm. Zoom on specific areas from WT of 16 months. Scale bar = 25 µm for (**b**) and 50 µm for (**c**, **d**). Representative pictures with *n* = 3 for each group. **b** Uncalcified articular cartilage (ac) and calcified cartilage (cc), separated by the tidemark (td—dotted line); chondrocytes (arrowheads). **c** Subchondral bone (sb), growth plate (gp) and lining cells (lc). **d** Metaphysis of the tibia showing the cortical bone (cb), the outer medial tibial side and the inner tibial side facing the bone marrow are indicated with (*)

We performed immunohistochemical detection of OMD in the knee joint of 4-, 8-, and 16-month-old mice. OMD was present at all ages in WT and UP mice but absent in KO mice, indicating the specificity of the immunostaining (Fig. 1a). OMD was strongly localized in the calcified cartilage ECM, while heterogeneous and light staining was also observed in the deep zone of the uncalcified articular cartilage ECM and in some chondrocytes (Fig. 1a, b). In bone, the lining cells were strongly stained as well as the ECM, mostly the mineralization front (Fig. 1a, c, d). The ECM and some cells in the meniscus were stained (Fig. 1a). The cartilaginous ECM of the growth plate was not stained (Fig. 1c). Immunostaining revealed that OMD is a proteoglycan with strong specificity for mineralized skeletal tissues.

### *Omd* influences bone and cartilage microarchitectures

#### Effect of *Omd* on articular cartilage structure

**Fig. 2:**
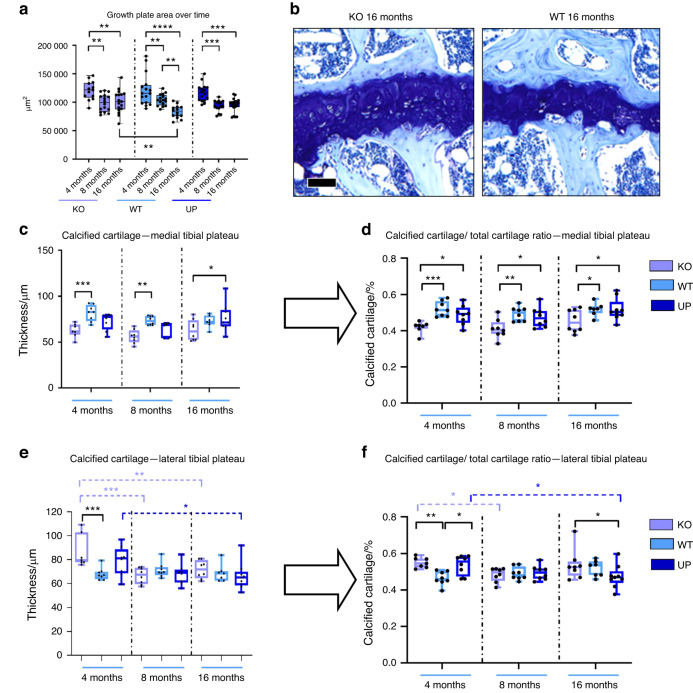
Histomorphometry of the cartilage was performed with QuPath at 4, 8, and 16 months. Knee joints of male mice were stained with Toluidine blue and areas corresponding to the total cartilage, the calcified cartilage, and the growth plate were measured for the medial tibial plateau and the lateral tibial plateau. **a** Measures of the growth plate area of both medial and lateral tibial plateaus were plotted to display the evolution of the growth plate over time with *n* = 13 for the KO, *n* = 16 for the WT and UP at 4 months; *n* = 16 for the KO and WT, and *n* = 15 for the UP at 8 months; *n* = 16 for the KO, *n* = 14 for the WT and *n* = 18 for the UP at 16 months. **b** Toluidine blue of the growth plate for the KO and the WT at 16 months are represented. Scale bar = 100 µm. **c**, **e** The thickness of the calcified cartilage was measured on the medial and lateral plateaus from the tibia. **d**, **f** The ratio between the calcified cartilage and the total cartilage two was reported for both the medial and lateral plateaus. For the medial plateau (**c**, **d**): *n* = 7 for the KO, *n* = 8 for the WT and UP at 4 months; *n* = 8 for the KO and WT and *n* = 7 for the UP at 8 months; *n* = 8 for the KO, *n* = 7 for the WT and *n* = 9 for the UP at 16 months. For the lateral plateau (**e**, **f**): *n* = 7 for the KO, *n* = 8 for the WT and UP at 4 months; *n* = 8 for each genotype at 8 months; *n* = 8 for the KO, *n* = 7 for the WT and *n* = 9 for the UP at 16 months. Two-way ANOVA was performed to evaluate the genotype effect (in black) and the time effect inside a genotype (in the corresponding color). The data were plotted as a box plot showing all points with differences being considered significant at *P* values <0.05 (*P < 0.05, **P ≤ 0.01, ***P ≤ 0.001, ****P ≤ 0.000 1)

Histological analysis revealed that the size of the tibial growth plate significantly decreased between 4 and 8 months in all genotypes but further decreased between 8 and 16 months only in the WT but not in other genotypes. At 16 months, the growth plate of the KO mice was larger than that of the WT mice (Fig. 2a, b).

In the 4-month-old KO mice, the calcified cartilage layer was thinner in the medial tibial plateau and thicker in the tibial lateral plateau than in the WT (Fig. 2c, e). The ratio of calcified cartilage/total cartilage for the medial tibial compartment of the KO mice was significantly lower than that in the WT and UP mice at each time point (Fig. 2d). In the tibial lateral plateau, this ratio was higher in the 4-month-old KO and UP mice than in the WT mice and in the 16-month-old KO mice than in the UP mice (Fig. 2f). Furthermore, this ratio decreased with age in the KO and UP genotypes, while it remained stable in the WT. The thickness of the cartilage (including uncalcified and calcified cartilage) was not different between genotypes except in the medial plateau of 8-month-old KO mice, in which the cartilage was thinner than in the WT (Fig. S2). Our data showed that *Omd* was able to influence the cartilage microarchitecture.

#### Effect of *Omd* on bone structure

##### Metaphysis of the tibia

**Fig. 3:**
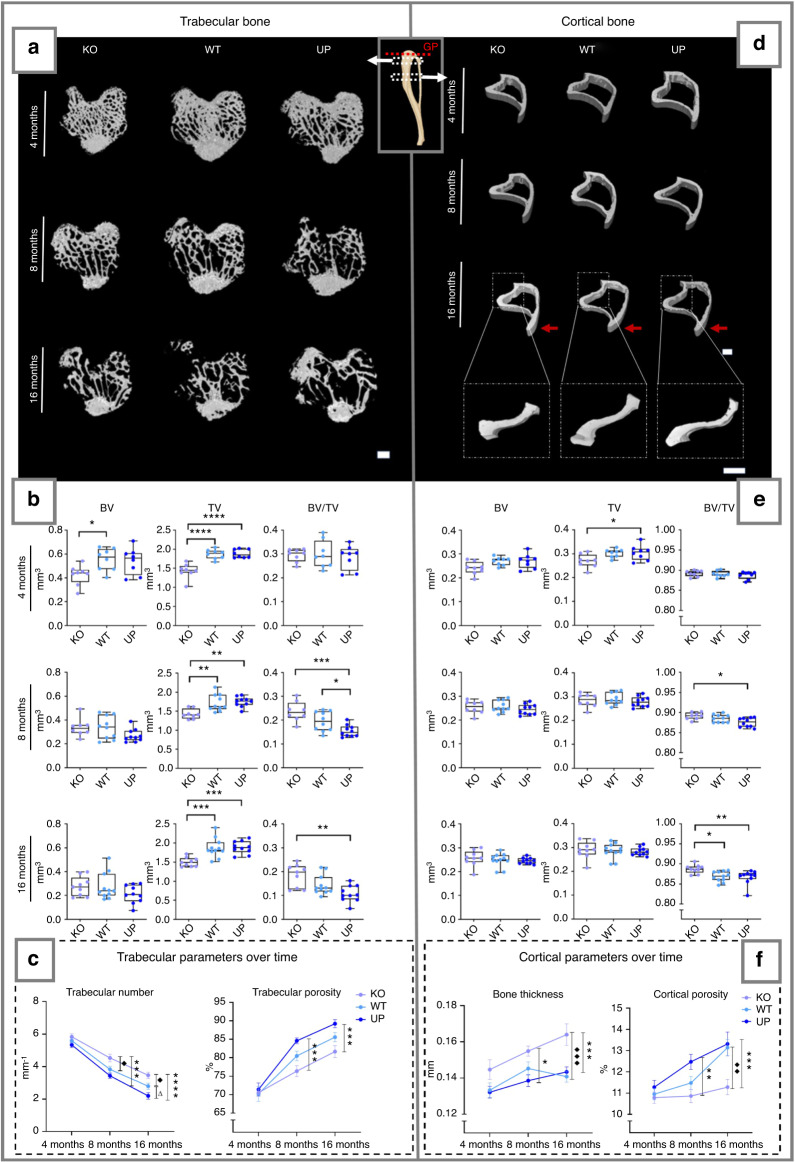
µCT analysis of the metaphysis of the tibia of the male mice at 4, 8 and 16 months. The trabecular bone (left) and the cortical bone (right) were analyzed separately. Regions measured for the trabecular bone and cortical bone are illustrated on the schematic tibia with the growth plate (GP), marked with a red dotted line, used as a reference for their selection. **a**, **d** The 3D rendering of each genotype is represented with a scale bar of 500 µm. Red arrows indicate the tibial crest on the 16 months cortical bone. The zoom on the 16 months cortical bone illustrates the lateral side of the tibia with a scale bar of 500 µm. **b**, **e** The bone parameters measured for the trabecular and cortical bone were the bone volume (BV); the total volume (TV) and their ratio (BV/TV). The data were plotted as a box plot showing all points. One-way ANOVA was performed with differences being considered significant at *P* values <0.05 (*P < 0.05, **P ≤ 0.01, ***P ≤ 0.001, ****P ≤ 0.000 1). **c**, **f** The trabecular number, trabecular porosity, cortical thickness, and cortical porosity are represented over time. At 4 months: *n* = 8 for each genotype; at 8 months: *n* = 8 for the KO, *n* = 10 for the WT and UP; at 16 months: *n* = 9 for the KO, *n* = 11 for the WT, and *n* = 10 for the UP. Two-way ANOVA was performed on the analysis over time with error bars representing ± SEM and differences being considered significant at *P* values <0.05, * represents significant differences between the KO and the UP, ♦ represents significant differences between the KO and the WT and Δ represents significant differences between the WT and the UP (*/♦/Δ*P* < 0.05, **/♦♦/ΔΔ*P* ≤ 0.01, ***/♦♦♦*P* ≤ 0.001)

The total volume of the trabecular bone was lower in the KO mice than in mice with other genotypes (Fig. 3a, b). The trabecular BV/TV ratio was not significantly different between genotypes at 4 months. In contrast, this ratio was significantly higher in the KO mice than in the UP mice at 8 and 16 months and lower in the UP mice than in the WT mice at 8 months (Fig. 3a, b). The number of trabeculae of the KO mice was higher at 8 and 16 months than that of the WT and UP mice, while no difference was observed at 4 months (Fig. 3c). The UP mice had significantly fewer trabeculae at 16 months compared to the WT. The porosity was lower in the KO and higher in the UP mice than in the WT at all ages. The porosity was significantly lower in the KO at 8 and 16 months than in the UP mice (Fig. 3c). The space between the trabeculae was greater in the UP than in the KO mice at 16 months, but no difference between genotypes was observed for the trabecular thickness. At 16 months, the structure model index of the UP mice was significantly higher than that of mice of another genotype, which indicated a shift from a plate to rod-like geometry of the trabecular bone (Fig. S3A).

In the cortical bone, the BV/TV ratio was higher in the KO mice than in the UP mice at 8 and 16 months and in the WT mice at 16 months (Fig. 3d, e). The cortical bone thickness increased with age in all genotypes but was significantly higher in the KO relative to the UP mice at 8 months and then relative to the WT and UP mice at 16 months (Fig. 3f). Cortical bone porosity was also affected by *Omd* expression. The porosity was consistently the lowest in the KO and the highest in the UP mice. The cortical bone porosity was significantly lower in KO mice than in UP mice at 8 and 16 months and then compared to that in WT mice at 16 months (Fig. 3c, f). In addition, the tibial crest was longer in KO mice than in WT and UP mice (Fig. S3B).

Both the loss-of-function and overexpression of *Omd* lead to interesting bone phenotypes. The KO mice had better conserved bone volume and had less porous bone, while the UP mice displayed more severe loss with a decrease in the trabecular number and an altered trabecular shape, and the WT mice adopted an intermediate phenotype. In addition, morphological changes have been reported between genotypes, with tibiae from the mutant not only being smaller and narrower but also showing a dissimilar shape as well as an extended tibial crest.

The microarchitecture modifications due to the loss-of-function of *Omd* reflected the bone’s physical properties. The biomechanical test showed a higher whole bone strength of the KO mouse tibia at 16 months, consistent with better bone volume conservation, which could endure a higher maximal load compared to WT and UP mice. The stiffness of the KO mice was also significantly greater than that of the UP mice at 16 months (Fig. S4).

##### Epiphysis of the tibia and the effect of Omd on the development of bone sclerosis

**Fig. 4:**
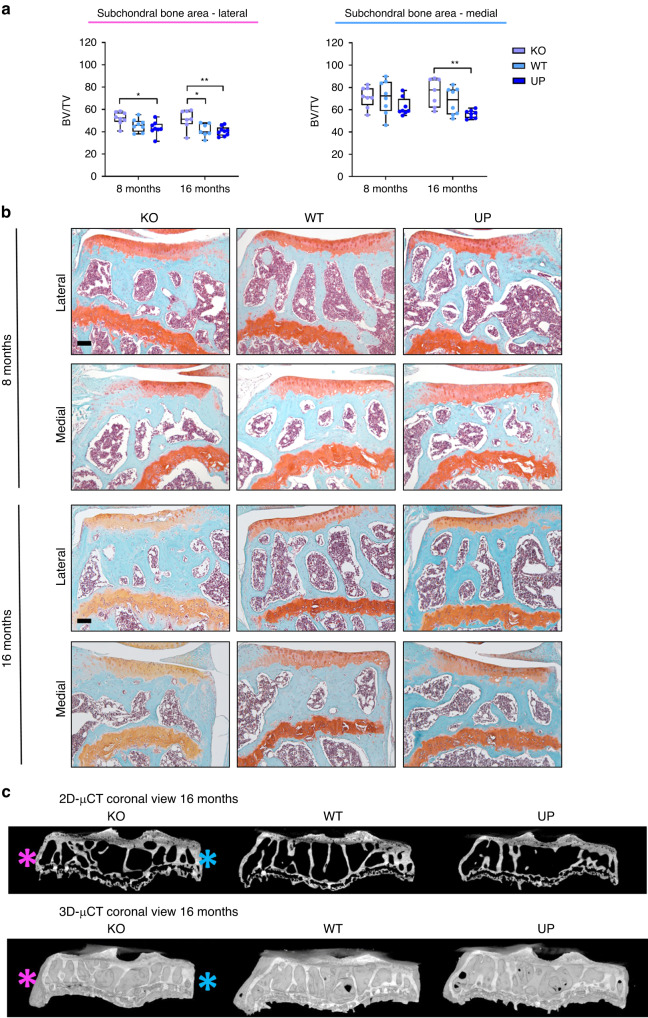
Effect of *Omd* on the spontaneous development of subchondral bone sclerosis. **a** Histomorphometry of the subchondral bone on Safranin-O Fast Green of the knee joint of male mice at 8 and 16 months was performed with QuPath on the lateral and medial plateaus of the tibia separately. At 8 months: *n* = 8 for each genotype; at 16 months: *n* = 7 for the KO, *n* = 7 for the WT, and *n* = 9 for the UP. The data were plotted as a box plot showing all points. Two-way ANOVA was performed with differences being considered significant at *P* values <0.05 (*P < 0.05, **P ≤ 0.01). **b** Representative picture of the Safranin-O Fast Green of the knee joint of male mice showing the subchondral bone area for the lateral and medial plateaus of the tibia separately. Scale bar = 100 µm. **c** µCT of the subchondral bone of the tibia of the mice at 16 months. The pink asterisk indicates the lateral plateau and the blue asterisk indicates the medial plateau

KO mice had a greater lateral subchondral bone BV/TV ratio than the UP mice at 8 and 16 months and compared to the WT at 16 months. In the medial tibial plateau, BV/TV was more elevated in KO mice than in UP mice but only at 16 months (Fig. 4a, b). Computed tomography illustrated that bone volume was higher in the KO mice than in the WT and UP mice (Fig. 4c).

*Omd* deficiency leads to a thicker bone at the tibial epiphysis and subchondral bone sclerosis. In contrast, overexpression of *Omd* by osteoblasts was associated with a decrease in bone volume. The differences between genotypes were clearly exacerbated in older mice.

### *Omd* may play a beneficial role against articular degradation and prevent subchondral bone sclerosis

**Fig. 5:**
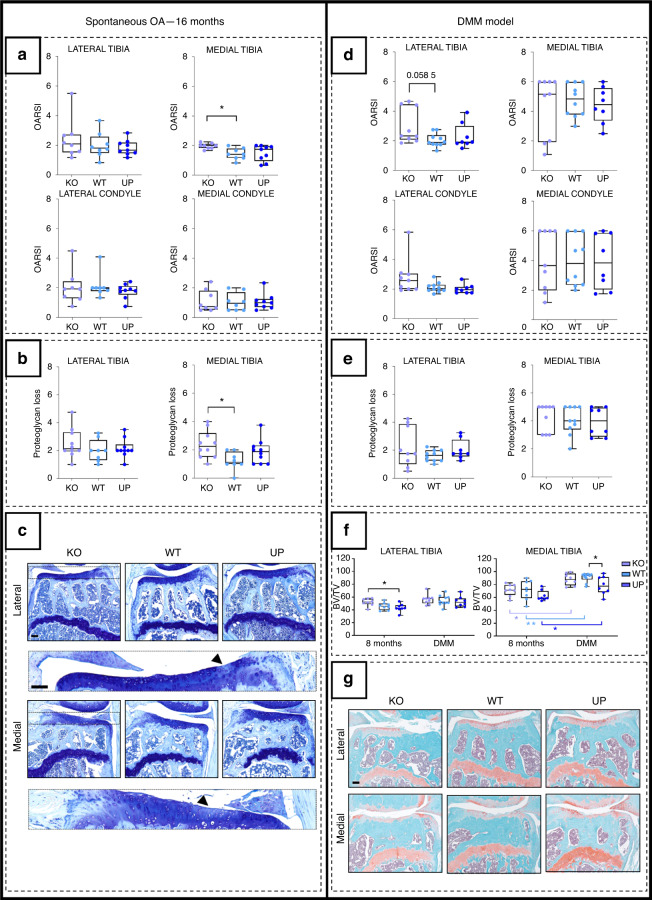
Analysis of the development of OA lesions in the different genotypes after spontaneously occurring with age (left) or after the DMM (right). The spontaneous OA lesions were considered in the 16-month-old male mice and the DMM was performed on 16-week-old male mice and they were stopped at 28 weeks. **a**, **d** The cartilage degradation was assessed with the OARSI score (from 0 to 6) according to the OARSI recommendations. The score was attributed to the lateral and the medial tibial plateaus and to the lateral and medial condyles for the spontaneous model and the DMM model. **b**, **e** The score of the loss of proteoglycan (from 0 to 5) was assessed according to the OARSI recommendations for the lateral and medial tibial plateaus for the spontaneous model and the DMM model. For the OARSI score of the 16-month-old mice: *n* = 8 for the KO and the WT and *n* = 9 for the UP and for the loss of proteoglycan *n* = 10 for the KO, *n* = 8 for the WT and *n* = 10 for the UP. For the DMM model: *n* = 9 for the KO, *n* = 10 for the WT, and *n* = 8 for the UP. One-way ANOVA was performed with differences being considered significant at *P* values <0.05 (*P < 0.05). **c** Illustrations of the lateral and medial plateaus stained with Toluidine blue of the 16-month-old mice with zooms on proteoglycan loss issued from the KO and indicated by the arrowhead. Scale bar = 100 µm. **f** Histomorphometry of the subchondral bone on Safranin-O Fast Green of the knee joint of the DMM mice was performed with QuPath on the medial and lateral plateaus separately. Each genotype was compared to a similar age group of 8-month-old mice. At 8 months: *n* = 8 for each genotype; for the DMM: *n* = 9 for the KO, *n* = 10 for the WT, and *n* = 8 for the UP. The data were plotted as a box plot showing all points. Two-way ANOVA was performed with differences being considered significant at *P* values <0.05 (*P < 0.05, **P ≤ 0.01). **g** Illustrations of the lateral and medial plateaus stained with Safranin-O Fast Green in the DMM. Scale bar = 100 µm

To study the role of *Omd* in the pathology of OA, we compared the spontaneous development of structural bone and cartilage changes in KO, WT and UP mice during aging and after destabilization of the medial meniscus. In nonoperated mice, cartilage lesions appeared with aging, and a higher OARSI score was observed in the medial tibial plateau of KO mice than in WT mice (Fig. 5a). This observation was consistent with the greater loss of proteoglycans in the medial tibial plateau of KO mice than in that of WT mice (Fig. 5b, c). No differences were observed in the lateral tibial plateau and femoral condyles in 16-month-old mice. In the DMM model, the lesions of the medial tibial plateau were severe, and no difference between genotypes was observed (Fig. 5d, g). Cartilage lesions were less severe in the lateral tibial plateau, and KO mice tended to have a greater OARSI score than WT mice (Fig. 5d, g), but the difference was not significant (*P* value = 0.058 5). No significant difference was observed for the scored loss of proteoglycans (Fig. 5e).

In WT mice with DMM-induced OA, the BV/TV ratio of the subchondral bone of the medial tibial plateau was significantly higher than that in nonoperated mice, while it was not affected in the lateral plateau. For the medial tibia, the source of variation analysis confirmed the highly significant effect of the surgery on the BV/TV ratio but also confirmed that the results were genotype dependent. The comparison of the medial subchondral bone BV/TV ratio between genotypes in DMM groups showed that it was lower in the UP mice than in the WT mice. Moreover, the UP mice from the DMM group displayed a BV/TV ratio remaining similar to that of the nonoperated WT mice. The two-way ANOVA comparison of the DMM model with the nonoperated mice showed no interaction between the two groups, which indicates that expected values are not related between both groups (Fig. 5f, g). These results suggest that the expression of *Omd* helps to prevent the development of subchondral bone sclerosis associated with OA.

*Omd* protects against the onset of subchondral sclerosis and may play a role in the prevention of subsequent articular damage, particularly for spontaneous OA.

### Loss of *Omd* expression induces gait abnormalities in mice

**Fig. 6:**
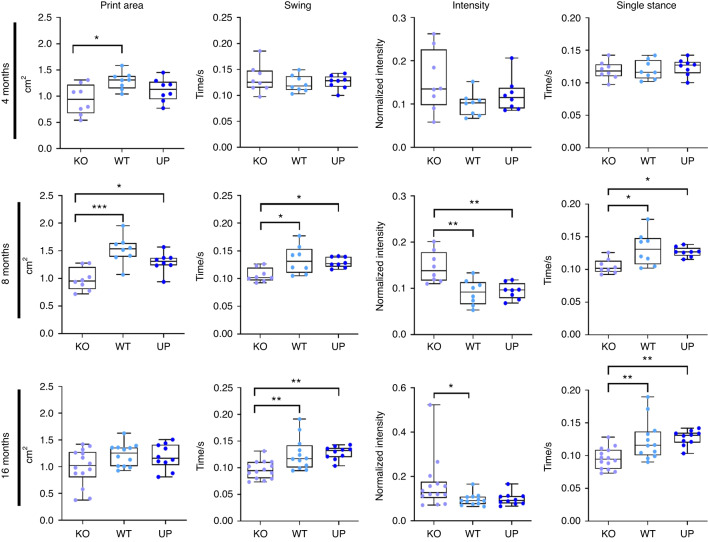
Analysis of the gait of 4-, 8-, and 16-month-old male mice with the CatWalk XT. The intensity corresponds to the mean intensity at the maximum paw contact normalized with the mean of the maximum contact paw area, the speed, and the weight of the mouse. At 4 months: *n* = 8 for each genotype; at 8 months *n* = 8 for each genotype; at 16 months: *n* = 14 for the KO, *n* = 12 for the WT and *n* = 10 for the UP. The data were plotted as a box plot showing all points. One-way ANOVA was performed when the distribution was Gaussian and Kruskal–Wallis was performed when the distribution was not Gaussian with differences being considered significant at *P* values <0.05 (*P < 0.05, **P ≤ 0.01, ***P ≤ 0.001)

The gait pattern of mice was assessed at all ages using the CatWalk XT platform. At 4 months, the print area was reduced in the KO mice compared to the WT mice, and at 8 months, the print area was smaller in the KO than in the WT and UP mice. The difference between the KO and other genotypes was no longer significant at 16 months, yet it is due to the reduced values of the WT and UP mice at 16 months, while the print area of the KO mice remained similar at each time-point. The swing, which is the duration of no contact of the paws with the walking platform, and the single stance, defined as the duration of contact of the paws with the walking platform, were shorter in the KO mice than in other genotypes at 8 and 16 months. Finally, the intensity of the contact of paws toward the glass platform was higher in the KO mice than in the WT mice at 8 and 16 months and compared to the UP mice at 8 months (Fig. 6 and Table S1).

The gait analysis clearly showed that KO mice have different gait behavior than other genotypes. Their abnormal gait could be explained by their distinct bone structure and worse cartilage degradation.

### *omd* is expressed in the zebrafish skeleton, and its mutation induces articular cartilage lesions and impaired bone remodeling

The zebrafish genome presents a single homolog to the human *OMD* gene, the ortholog *omd* encoding a 401 amino acid protein presenting 46% identical and 63% similar amino acids.

We characterized the localization of *omd* expression in larval zebrafish using whole-mount in situ hybridization at 48 h post fertilization (hpf), 5 days post fertilization (dpf) and 8 dpf. We observed strong expression of *omd* specific to craniofacial cartilage, including the jaw joint, during development (Fig. S5).

To gain first insights into the function of *omd* in zebrafish cartilage development, we studied the overexpression of *omd* by microinjecting 0.4 ng and 0.8 ng of its mRNA into zygotes. At 24 hpf, *omd* induced ventralization of embryos that was not observed upon microinjection of control GFP mRNA (Fig. S6A, B). Furthermore, larvae injected with *omd* developed deformities at 4 dpf, mostly affecting axial symmetry. Larvae presenting axial deformities demonstrated evident cartilage defects with abnormal development of the craniofacial cartilage (Fig. S6C). As *omd* overexpression induced developmental defects, we designed a zebrafish *omd* mutant line (*omd*^*−/−*^) for further characterization in adults. In situ hybridization of *omd*^*−/−*^ individuals revealed that the mutation led to the absence of *omd* mRNA in the craniofacial structures (Fig. S5), indicating that no Omd protein was produced in the mutants.

**Fig. 7:**
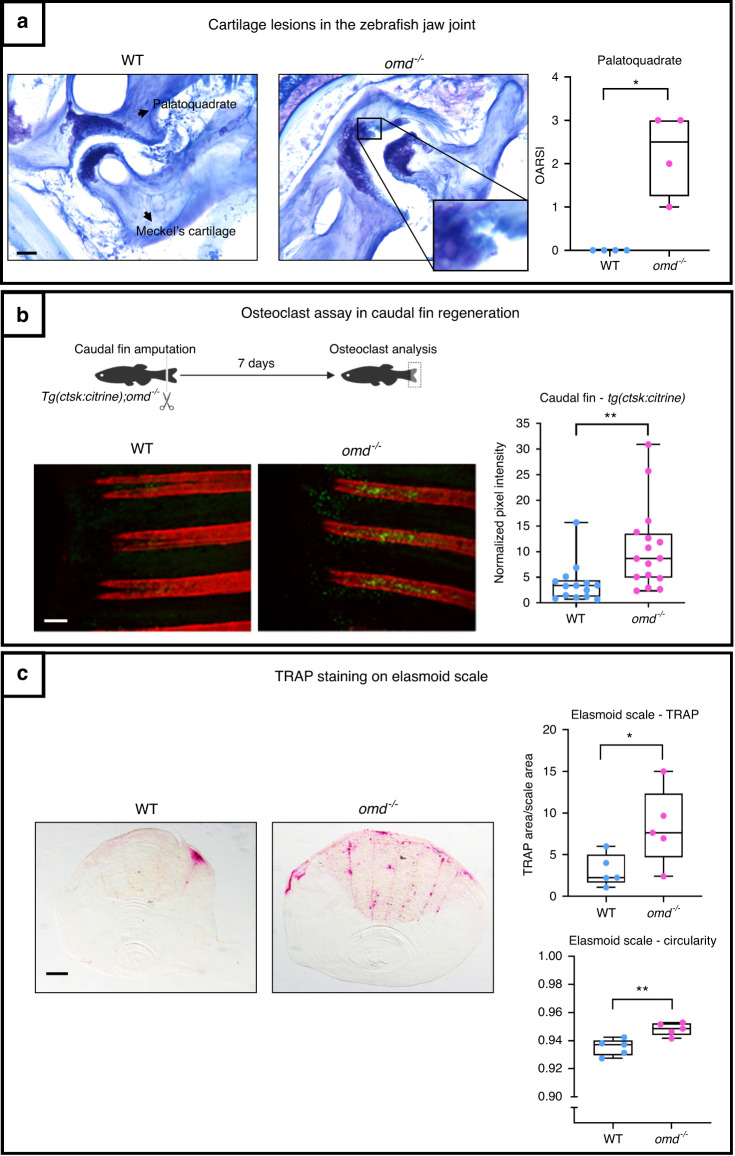
The mutant deficient for *omd* was generated through CRISPR/Cas 9. **a** Histology of the jaw joint was performed on 1-year-old zebrafish. The OARSI score of the palatoquadrate was attributed to the jaw joint stained with Toluidine blue with *n* = 4 for the WT and the mutant. The data were plotted as a box plot showing all points. Mann–Whitney test was performed with differences being considered significant at *P* values <0.05 (*P < 0.05). Scale bar = 200 µm. **b** The mutant line was crossed with the *Tg(ctsk:Citrine)* for the osteoclasts analysis during the caudal fin regeneration. The caudal fin of 1-year-old zebrafish was cut and the regenerating fin was observed after 7 days. The osteoclasts are represented in yellow-green from the ctsk:Citrine signal and the mineralized ray were stained with Alizarin red. The data were plotted as a box plot showing all points. The pixel intensity of the regenerating rays is plotted and normalized by the background intensity with *n* = 14 for the WT and *n* = 16 for the mutant from two independent experiments which were pooled to perform the unpaired Student’s *t* test with differences being considered significant at *P* values <0.05 (**P ≤ 0.01). Scale bar = 200 µm. **c** TRAP staining was performed on the elasmoid scales of 1.6-year-old zebrafish. The TRAP staining area was normalized with the total scale area. The TRAP staining and the circularity of the scales were assessed with ZFBONE—Fiji with *n* = 5 for the WT and the mutant and with 6 to 15 scales/zebrafish analyzed. Scale bar = 0.2 mm. The data were plotted as a box plot showing all points. Unpaired Student’s *t* test with differences being considered significant at *P* values <0.05 (*P < 0.05, **P ≤ 0.01)

We then compared the lubricated synovial jaw joints in 1-year-old *omd*^*−/−*^ zebrafish to those in WT zebrafish to detect articular cartilage damage. For the palatoquadrate, the OARSI score of mutants (ranging from 1 to 3) was greater than that of the WT, and clefts on their articular cartilage were observed (Fig. 7a). As the murine model showed that the level of expression of *Omd* was related to the onset of subchondral bone sclerosis and that zebrafish display lubricated synovial joints with similar articular degradation and subchondral bone modifications as in the physiopathology of OA,^32,33^ we took advantage of zebrafish to assess osteoclasts in vivo. We used this model to investigate the expression of cathepsin K, a marker of osteoclasts, in the regenerating caudal fin at 7 days postamputation, a condition associated with osteoclastogenesis. Cathepsin K expression was significantly higher in *Tg(ctsk:Citrine); omd*^*−/−*^ zebrafish, indicating that more osteoclasts were generated in the absence of *omd* expression (Fig. 7b). Furthermore, osteoclast activity was studied in elasmoid scales through TRAP staining. More TRAP staining was present on the elasmoid scale of *omd*^*−/−*^ zebrafish. The staining appeared to be more evenly distributed throughout the scales of the mutant and particularly localized on the edges and along the grooves of the scale. The circularity of the scales was also impacted. The scales of the *omd*^*−/−*^ zebrafish were more circular than those of the WT zebrafish (Fig. 7c).

Our observations of the zebrafish confirmed our observations of the mouse model. The *omd* mutant zebrafish showed more severe spontaneous articular cartilage degradation in the synovial jaw joint. They also demonstrated that the regulation of osteoclastogenesis was a possible mechanism of action for Omd.

### OMD inhibits osteoclastogenesis by binding to RANKL

We investigated the effects of OMD treatment on gene expression in cultured human primary trabecular osteoblasts. RNA-seq revealed that only 35 genes (with *P*adj < 0.05) were differentially expressed after 10 ng·mL^−1^ OMD treatment, with relatively modest changes in expression (Fig. S7 and Table S2). GSEA using WebGestalt on GO terms revealed an increase in some genes linked to the response to acidic chemicals (genes *AKR1C1*, *AKR1C2*, *AKR1C3*) and a decrease in a few genes involved in extracellular structure organization (GO:0043062) and ossification (genes *ACAN* and *IBSP*) and in the molecular function of actin binding. The Reactome database revealed an upregulation of genes responsible for collagen network degradation and a downregulation of ECM proteoglycans, ECM organization, collagen formation, and integrin cell-surface interactions.

**Fig. 8:**
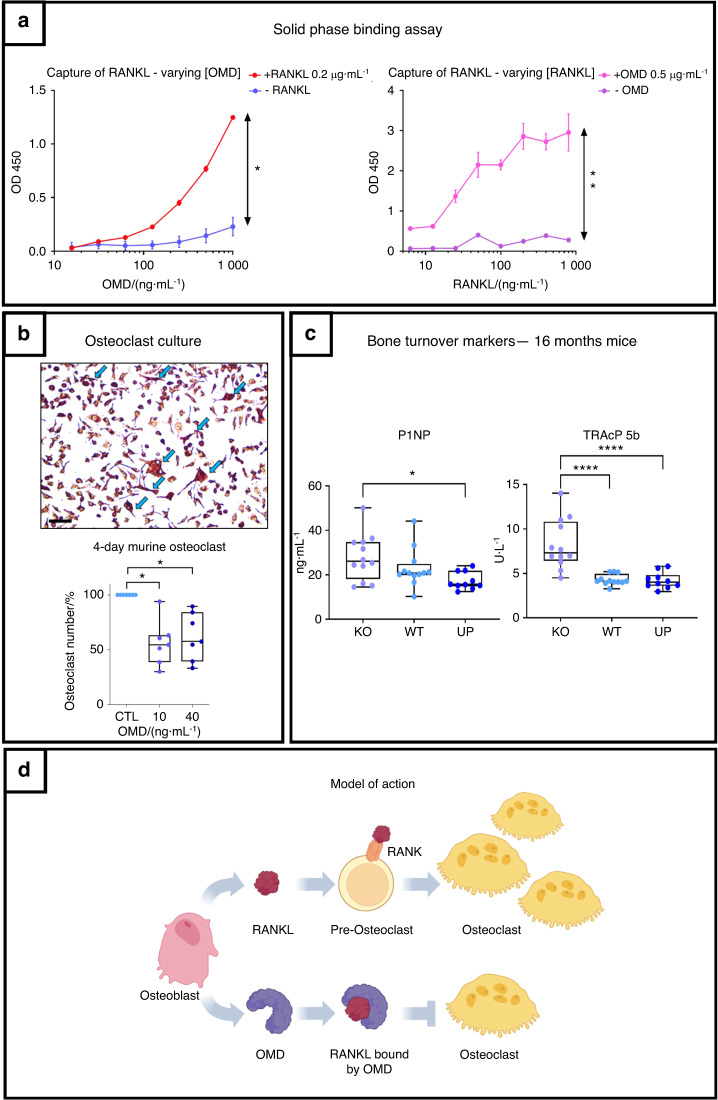
OMD inhibits osteoclastogenesis through its direct interaction with RANKL and balances bone remodeling. **a** Solid phase binding assay on the capture of RANKL by OMD. RANKL was coated on a plate followed by OMD addition. On the left: Binding assay with different concentrations of OMD (1 000 to 15.65 ng·mL^−1^ by serial 2X dilution), with 0.2 μg·mL^−1^ of coated RANKL (red curve) and negative control without RANKL (blue curve). On the right: Binding assay with different concentrations of coated RANKL (800 to 6.25 ng·mL^−1^) and 0.5 μg·mL^−1^ of given OMD (pink curve); negative control without OMD (purple curve). Wilcoxon test was performed with differences being considered significant at *P* values < 0.05 (*P < 0.05, **P ≤ 0.01). **b** Assay of the effect of OMD on primary murine osteoclast culture. The peripheral blood mononuclear cells were collected from murine bone marrow and differentiated into osteoclasts with M-CSF and RANKL. Osteoclasts were counted after 4 days of differentiation following a TRAP staining. Each point represents a mouse, *n* = 7. The osteoclast count was represented in percentage of cells with the corresponding control set as 100%. Blue arrows point at osteoclasts. Scale bar = 50 µm. The data were plotted as a box plot showing all points. One-way ANOVA was performed with differences being considered significant at *P* values <0.05 (*P < 0.05). **c** Level of P1NP and TRAcP 5b measured in the serum of KO, WT and UP mice. The data were plotted as a box plot showing all points with *n* = 12 for the KO and WT, and *n* = 10 for the UP. One-way ANOVA was performed when the distribution was Gaussian and Kruskal–Wallis was performed when the distribution was not Gaussian with differences being considered significant at *P* values < 0.05 (*P < 0.05, **P ≤ 0.01, ****P ≤ 0.000 1). **d** Schematic representation of the mechanism of OMD on osteoclastogenesis. Osteoblasts secrete RANKL which binds to the RANK receptor on the membrane of pre-osteoclasts to induce their differentiation into osteoclasts. In parallel, osteoblasts also secrete OMD which displays the ability to capture RANKL and would prevent its binding to RANK

These observations led us to explore the interaction of OMD with RANKL, which is the regulator of osteoclast differentiation, using a solid phase binding assay. These experiments revealed a clear interaction between the two proteins, which correlated with the increasing amounts of both OMD and RANKL, demonstrating that they bind directly to each other (Fig. 8a). The potential biological effects of this interaction were tested in primary murine osteoclast culture. We showed that OMD added at 10 and 40 ng·mL^−1^ reduced osteoclast number. No difference between 10 and 40 ng·mL^−1^ of OMD was observed, both reducing the osteoclast number by 50% on average (Fig. 8b). Furthermore, serum markers for bone formation (N-terminal propeptide of type I procollagen: P1NP) and osteoclast number (TRAcP 5b) showed impaired bone turnover in the KO mice at 16 months. The serum level of P1NP was significantly higher in the KO mice than in the UP mice, and the KO mice showed significantly increased TRAcP 5b compared to the WT and UP mice (Fig. 8c).

We propose a model in which osteoblasts secrete OMD in the ECM to trap RANKL and prevent it from binding to the preosteoclast receptor RANK to inhibit their differentiation into fully committed osteoclasts. In this model, OMD depletion induces uncoupled bone remodeling, in which increased osteoclast number and bone resorption, further associated with the stimulation of bone formation, lead to subchondral bone sclerosis (Fig. 8d).

## Discussion

OMD is a small proteoglycan involved in bone and dental matrix mineralization but also in ectopic mineralization of other tissues, such as arteries,^34–38^ suggesting that it could be involved in cartilage mineralization and degradation during aging and OA.

We previously demonstrated that osteoblasts located in the sclerotic area of OA subchondral bone produced less OMD than neighboring osteoblasts from the nonsclerotic area. Interestingly, OMD levels were also lower in the serum of OA patients.^8^ To study the impact of *Omd* expression on bone remodeling, skeletal development and architecture, we followed mice deficient in *Omd* and mice overexpressing *Omd* for 16 months.

While the presence of OMD in bone has been previously reported,^26,30^ we showed for the first time that OMD is localized in mineralized tissues of the murine knee joint and is identified in calcified cartilage and tidemarks. Interestingly, we observed that the calcified cartilage layer was thinner in the medial tibial compartment but thicker in the lateral tibial compartment of KO mice than in other genotypes, indicating that OMD plays a key role in cartilage mineralization. The consequences of calcified cartilage thickness on cartilage degradation in OA remain controversial. One study showed that the calcified cartilage was thinning with OA, resulting in a reduction in the cartilage elastic modulus.^39^ However, other studies showed that the calcified cartilage thickness increased with the progression of OA.^40,41^ The presence of more severe cartilage lesions in the medial tibial plateau of aging KO mice, where the calcified cartilage was thinner, supports the hypothesis that a thinner layer of calcified cartilage is a factor promoting cartilage degradation. Of course, this theory needs to be confirmed in other models. In the DMM OA model, there was no significant difference in cartilage damage between genotypes. This finding contrasted with the observation made in the aging KO mice, in which the cartilage lesion severity was higher in KO mice than in mice of other genotypes. This observation can be explained by the higher severity of the lesions in the DMM model reflecting more of a late stage of OA. We can anticipate a ceiling effect in the DMM-induced OA model because the cartilage lesions were too severe. However, considering that most of the phenotypic changes induced by the modification of the expression of *Omd* worsened with age, the experimental design of the DMM model does not make it possible to exclude an effect of *Omd* on the articular cartilage degradation occurring at the stage set for the surgery.

At the bone level, 8- and 16-month-old KO mice had greater trabecular and cortical BV/TV than the WT mice, while inversely, UP mice had a reduced ratio. This finding highlights that *Omd* plays a key role in bone remodeling. More precisely, maintaining the homeostatic expression of *Omd* helps to preserve its volume and structure. *Omd* overexpression not only reduced BV/TV but also increased the structure model index, which is an indicator of the altered shape of trabeculae, and led to higher bone porosity. Over time, aging aggravated those observations. This indicates that when overexpressed, *Omd* may cause detrimental effects on skeletal tissues. In the KO, the global bone morphology was affected. Their tibiae were narrower, and their tibial crests were longer. This morphological change may affect muscle insertion and, therefore, the muscle-to-bone relationship. Furthermore, KO mice were more prone to spontaneously develop subchondral bone sclerosis, as indicated by higher BV/TV, similar to sclerotic subchondral bone in OA. The modifications of the bone microarchitecture affected the bone’s physical properties as well, important features relative to the bone quality, with KO mice showing higher whole bone strength of the tibia at 16 months, an expected finding following the observations from the µCT analysis.

We also observed sclerosis of the subchondral bone following the DMM procedure in all genotypes. However, the subchondral bone of the medial tibia of the UP mice was thinner than that of the KO and WT mice, suggesting that *Omd* could prevent subchondral bone sclerosis in OA. Although these observations indicate that the overexpression of *Omd* would protect against the onset of OA-associated sclerosis, we are still willing to be cautious about the efficiency of the protective effect, as the BV/TV ratio is initially slightly lower in the UP mice showing altered bone microarchitecture. Nonetheless, our data suggest that *Omd* plays a role in subchondral bone sclerosis, a key feature of OA, which is involved in cartilage degradation. Therefore, we hypothesize that the impact of *Omd* on cartilage degradation could be secondary to its effect on bone. In the spontaneous model, the loss-of-function of *Omd* was associated with articular cartilage degradation, suggesting that *Omd* might prevent cartilage degeneration. This hypothesis has to be verified in a larger number of animals or a more advanced aging-related OA model where more pronounced differences with a higher articular degradation in the KO mice are anticipated.

Gait analysis with Catwalk XT identified different motor patterns between the genotypes. The gait pattern of KO mice, including the print area, swing, intensity of contact, and single stance of paws toward the glass platform, was modified. More precisely, for their hind paws, the KO mice had a reduced print area, a shorter swing and single stance, and a higher intensity of the contact of the paw. This may result in pain, discomfort, or mechanical disorders associated with joint damage or skeletal tissue abnormalities. It is important to highlight that a decreased hind print area is considered the best predictor for spontaneous OA.^42^ Furthermore, the single stance was reported to be significantly decreased in OA mice,^43^ and the swing may also be reduced with OA.^44^ Our report on gait parameters is consistent with previous observations found in the literature and corroborates the susceptibility of KO mice to develop further OA joint damage. On the other hand, the gait patterns at 16 months may be affected by the initiation of OA, leading to abnormal loading on affected limbs.

To corroborate our findings from the mouse model, we studied mutant adult zebrafish that did not express *omd*. In zebrafish, we found cartilage lesions in the articular cartilage in the jaw joint. Again, this suggests that *omd* prevents spontaneous cartilage lesions during aging and that a decrease in OMD production by osteoblasts and hypertrophic chondrocytes could be deleterious for cartilage. Altogether, these findings support the idea that a loss of OMD contributes to OA development. We then investigated by which mechanism of action OMD could regulate bone and cartilage metabolism. Our transcriptomic data revealed that OMD is unlikely to perform its function on osteoblasts through direct gene expression regulation, as very few genes were modified and with a low magnitude. It remains noteworthy to specify that among the regulated genes, *IBSP* was downregulated by OMD. *IBSP* overexpression by hypertrophic chondrocytes is associated with OA.^45^ Consequently, OMD could control cartilage calcification in OA by downregulating IBSP production.

Direct binding of OMD to key bone regulatory factors is another possible mechanism of action. SLRPs are known to bind cytokines, growth factors, and ligands such as RANKL.^9,24^ Herein, we showed that OMD not only enhances the differentiation of osteoblasts^30^ but is also able to bind directly to RANKL and block its biological activity on osteoclasts. Measures of bone markers in the serum of 16-month-old mice corroborated the elevated osteoclast number and higher bone turnover in the loss-of-function model. The UP mice showed the lowest P1NP level, which might indicate that their poor conservation of bone volume was due to low bone formation rather than bone resorption. The mutant zebrafish model confirmed the role of *omd* in osteoclastogenesis. The number of cathepsin K-positive osteoclasts increased in the regenerating caudal fin of the mutants. Furthermore, observations of elasmoid scales, which share a similar transcriptomic profile with the mammalian skeleton, including genes related to human diseases,^46^ highlighted higher TRAP staining and more circular scales in zebrafish lacking *omd*. As osteoclasts induce *Omd* expression in mature osteoblasts,^31^ OMD exerts negative feedback regulation on them. Moreover, sulfated GAGs are known to inhibit the differentiation of osteoclasts, and the sulfation level of OMD is higher during the ECM mineralization process.^47,48^ Our findings present OMD as a novel regulator of the bone remodeling process that is able to protect against subchondral bone sclerosis in pathological conditions such as OA. These observations are crucial since we know that bone remodeling plays a key role in the bone-driven OA phenotype, with uncoupled bone remodeling leading to impaired bone resorption and bone deposition functions, resulting in subchondral bone sclerosis with an elevated number of osteoclasts.^49–51^ We identified OMD as a novel player in the uncoupled bone remodeling associated with OA. Finally, we can point out that the bone and cartilage phenotypes due to the loss-of-function of *Omd* are consistent with other murine SLRP-deficient models.^16–24^ This ultimately reinforces previous statements declaring overlapping functions within the SLRP family.

In conclusion, alterations in *Omd* expression modify bone and cartilage metabolism and structure. OMD helps to preserve bone and cartilage integrity, and a local decrease in its production leads to the development of OA mainly by increasing subchondral bone sclerosis and thinning the calcified cartilage, while its overexpression alleviates subchondral bone sclerosis. OMD is able to directly bind to RANKL and inhibit osteoclastogenesis to regulate bone remodeling and limit subchondral bone sclerosis. Our previous and current studies, making use of both in vitro and in vivo experiments either with human, mouse or zebrafish models, build a strong and compelling body of evidence that OMD is a key factor in OA associated with subchondral bone sclerosis.

## Methods

### Mouse strains and housing

The mutant mouse strain deficient for *Omd* used for this research project, C57BL/6 *Omd*^*tm1Lex*^*/Mmucd*, RRID: MMRRC_011749-UCD, was obtained from the Mutant Mouse Resource and Research Center (MMRRC) at the University of California at Davis, an NIH-funded strain repository, and was donated to the MMRRC by Lexicon Genetics Incorporated. The mutation targeted coding exons 1 and 2 by homologous recombination. The genotyping protocol from MMRRC was applied. The overexpressing mouse strain for *Omd* used for this research project, C57BL/6 *Tg(Bglap-Omd)1Kieg*, EMMA ID EM:02120, was obtained from the European Mouse Mutant Archive (EMMA), a repository supported by the national research programs and by the EC’s Research and Innovation program Horizon 2020. The transgenic line expressed *Omd* under the osteocalcin promoter in addition to its natural expression; hence, *Omd* overexpression was only osteoblast specific. Strains were crossed with WT C57BL/6 mice to maintain the line. Transgenic, WT, and mutant mice were maintained on a 12-h light/dark cycle with food and water supplied ad libitum. To simplify the nomenclature in the paper, we refer to the *Omd*-deficient mice as “KO” and to the *Tg(Bglap-Omd)* as “UP”. The ethics committee of the University of Liège approved all experimental procedures (reference no. 19-2090).

### Mouse model of OA

Posttraumatic OA was induced by DMM in the UP, WT, and KO strains at 16 weeks. The surgical transection of the medial menisco-tibial ligament of the right knee was performed to induce mild instability of the knee.^52^ The mice were euthanized 12 weeks after surgery, and their knees were histologically analyzed.

For spontaneous OA, UP, WT, and KO mice were euthanized at 16 months, and their knees were histologically analyzed.

### Knee joint histology and histomorphometry

Knee joints of the mice, at 4, 8, 16 months, and 28 weeks from the DMM model, were fixed for 24 h in 4% paraformaldehyde (PFA) at 4 °C, followed by decalcification in hydrochloric acid (DC2 medium; Labonord) for 2 h and 30 min at 4 °C and then washed in Milli-Q water overnight at 4 °C before embedding in paraffin. Coronal sections of 5 μm were cut within the central area with 3 sections at least 80 μm apart selected for analysis with Safranin-O Fast Green staining. An additional central section was used for Toluidine blue staining. Each compartment of the knee joint was scored by two readers following OARSI guidelines for the mouse model as described in ref, ^53^ and the mean score from the 3 sections was calculated.

Histomorphometry of the sections was performed with QuPath version 0.3.2 software.^54^ The sections were photographed at ×10 magnification. Cartilage histomorphometry analysis was performed on sections stained with Toluidine blue. The total cartilage, calcified cartilage, plate length, and growth plate area were measured. For the growth plate, the area was measured inside a consistent circle of a fixed size under the articular plateau. The subchondral bone area analysis was performed on the 3 sections stained with Safranin-O Fast Green. The bone area was measured under the tibial plateau according to its length, and the bone marrow area was removed. The measured region of interest (ROI) is explained in Fig. S8.

### Immunohistochemistry

Epitope retrieval was performed using chondroitinase ABC (50 units per mL, Sigma-Aldrich) in 60 mmol·L^−1^ sodium acetate and 100 mmol·L^−1^ Tris (pH 8) for 30 min at 37 °C. Animal-free blocking solution (Cell Signaling Technology, dilution 5X) was used to block the sections prior to overnight incubation with the primary polyclonal goat antibody anti-mouse OMD (R&D Systems, AF3308, 0.8 μg·mL^−1^) in antibody diluent (Dako, S2022). The sections were then incubated for 30 min with the secondary polyclonal rabbit antibody anti-goat coupled with HRP (DakoP0449, dilution 1:400) diluted in Antibody diluent. Visualization of the secondary antibody was performed by using DAB (Cell Signaling Technology, 8059) for 2 min. Sections were counterstained with hematoxylin from Carazzi (Sigma-Aldrich) for 4 min.

### Microcomputed tomography (µCT) and image analysis

Tibiae from mice were dislocated and fixed for 24 h in 4% PFA at 4 °C and transferred into phosphate-buffered saline (PBS) for storage at 4 °C. Samples were imaged using a Phoenix NanoTom M (GE Measurement and Control Solutions, Germany). A diamond target was applied, and scans were operated at a voltage of 60 kV, a current of 170 μA, and a voxel size of 3 µm. An aluminum filter of 0.2 mm was used to reduce beam hardening during the acquisition. The exposure time was 500 ms, and 1 800 images were acquired over 360° using the fast scan mode (frame averaging = 1; image skip = 0). During reconstruction (Datos|x, GE Measurement, and Control Solutions), we applied a beam hardening correction of 8. After reconstruction, scans were oriented in the same plane using DataViewer (Bruker MicroCT, Kontich, Belgium). Images were analyzed using CTAn (Bruker MicroCT, Kontich, Belgium). For assessment of the trabecular architecture, we selected 150 images (450 µm height) starting at 30 μm below the growth plate level. Using 3D analysis, the trabecular volume (BV), total ROI volume (TV), number of trabeculae, porosity, and structure model index (SMI) were calculated. For analysis of the cortical architecture, we selected 100 images (300 µm height) starting at 1 500 μm below the growth plate level and corresponding to the mid-shaft. Using 3D analysis, the BV, TV, cortical thickness, porosity and tibial crest length were calculated. 3D visualization was performed using CTVox (Bruker MicroCT, Kontich, Belgium). The subchondral bone of the tibia, showing a coronal view of the medial and lateral plateaus, was also visualized in 3D using CTVox, and a 2-D visualization was generated using DataViewer.

### CatWalk XT

The gait analysis of the mice was performed using the CatWalk XT System (Noldus, Netherlands; software version XT 10.5). The CatWalk XT platform was placed in a dark and silent environment to enhance the quality of the recording and reduce animal stress. The same detection settings were used for each mouse: camera gain of 18.99 dB, green intensity threshold of 0.1, detection threshold of 0.1 a.U, red ceiling light of 17.2 V, and green walkway light of 16.5 V. The gait was recorded, and the CatWalk XT software automatically labeled the footprint and generated the various associated gait parameters for the compliant runs. A compliant run was defined as a run where the mouse did not stop while going through the walkway with at least 12 footprints, the maximum variation was set at 60%, and the speed was between 10 and 45 cm·s^−1^. At least 3 runs for each mouse were recorded, and the data represent the mean value. The data from the left and right paws were pooled for the front and hind paws to simplify the run parameter visualization.

### Zebrafish husbandry and strains

Zebrafish (*Danio rerio*) were raised in standard conditions as described in ref. ^55^ Mutant lines deficient for *omd* were generated using CRISPR‒Cas9 mutagenesis with the guide RNA *5’-*CAA-GAG-CTG-CGC-CAA-TG-TCA-3’. The gRNAs targeting *omd* were incubated with Cas9 protein (Thermo Fisher Scientific) before microinjections into 1-cell stage zygotes. The mutation targeted the START codon. The reporter line used to visualize osteoclasts is the transgenic line *TgBAC(ctsk:Citrine)*^56^ and was kindly provided by Prof. Stefan Schulte-Merker. The ethics committee of the University of Liège approved all experimental procedures (references no. 16-1961 and 19-2133).

### Injection of mRNA of *omd* in the Zebrafish

For *omd* overexpression, zebrafish *omd* mRNA and *GFP* mRNA, serving as a control, were microinjected into 1-cell stage zygotes. The following primers were used to generate the *omd* mRNA: forward *5’-CGA GAG AGA TAT TCA ATC CCA CAG-3’* and reverse *5’-TCA ACC AAC AAG GAA TGG AAG-3’*. The T7 promoter sequence for in vitro mRNA synthesis with the mMessage mMACHINE®T7 Ultra kit (Invitrogen) was added afterward with nested PCR using the forward primer *5’-GCG AAT TGT AAT ACG ACT CAC TAT AGG GCC ACC ATG ACA TTG GCG CAG-3’*. Fertilized eggs were injected with either 0.4 ng or 0.8 ng of mRNA. Phenotypic characterization was performed at 24 hpf and 4 dpf. At 4 dpf, the larvae were fixed with 4% PFA overnight at 4 °C and then stained with Alcian blue as described in ref. ^57^

### Whole-mount in situ hybridization in zebrafish

Zebrafish larvae at 48 hpf, 5 dpf and 8 dpf were used for whole-mount in situ hybridization. Larvae were raised in the presence of 0.003% 1-phenyl-2-thiourea until 5 dpf to avoid pigmentation development. Larvae were fixed overnight in 4% PFA at 4 °C and stored in 100% methanol at −20 °C until use. Visible in situ hybridizations were performed as described in ref. ^58^ with a digestion step with Proteinase K (Thermo Scientific) at 40 μg·mL^−1^ for 30 min at 37 °C for the 48 hpf larvae, at 50 μg·mL^−1^ for 30 min at room temperature, and at 40 μg·mL^−1^ for 50 min at room temperature for the 8 dpf larvae.

### Histology of the zebrafish jaw joint

One-year-old zebrafish were fixed with 4% PFA at 4 °C for a minimum of 24 h and decalcified in 1 mol·L^−1^ EDTA solution for 20 days. Zebrafish were dehydrated in ethanol, embedded in paraffin, and sagittally sectioned at 5 µm. Sections showing the jaw joint were stained with Toluidine blue. The OARSI score was attributed to 1 section per jaw joint as described in ref. ^32^

### Zebrafish osteoclast assay in the caudal fin

The *omd* x *TgBAC(ctsk:Citrine)* mutants were used at 1 year for the osteoclast analysis. Their caudal fins were cut, and the fins were allowed to regenerate for 7 days. Regenerated caudal fins were cut for analysis and incubated for 20 min with 0.01% Alizarin red S (Sigma‒Aldrich) to stain the mineralized bone matrix. Quantification of fluorescence from regenerated rays was performed using ImageJ software.^59^

### TRAP staining of the zebrafish scales

Ontogenetic scales of 1.6-year-old fish were plucked from the flank of the zebrafish and fixed with 4% PFA at room temperature for 30 min. Scales were incubated for 2 h in the TRAP staining solution as described in ref. ^60^ Quantification of TRAP staining was performed using ZFBONE software on FIJI.^61^

### Human trabecular osteoblast culture and RNA-seq analysis

Tibial bones were obtained from 6 male and 5 female patients undergoing total knee replacement surgery for OA. The age of the patients ranged from 58 to 89 years. All tissue samples used in this study were obtained after receiving approval from the University of Liege Medicine Faculty ethics committee (No. B70720108313, reference 2010/43), and written informed consent was obtained from each subject. Nonsclerotic trabecular bone was easily removed from the tibia with surgeon pliers and enzymatically processed to obtain digested bone pieces cultured as described in ref. ^62^ At confluence, osteoblasts were collected by trypsinization and seeded (22 000 cells per cm^2^) in 12-well plates (Nunc). Osteoblasts were cultured until confluence and then switched into differentiation media as described in ref. ^62^ for 3 days in the presence of 10 ng·mL^−1^ human recombinant OMD (R&D Systems, 2884-AD) or its absence for the same patient, serving as its own control.

Total RNA was extracted from osteoblast cultures, with RNA quality indicator scores (RIN) of 9.3, and RNA-seq for differential gene expression analyses was performed with a false discovery rate (FDR) of 0.01 to assess the statistical significance as described in ref. ^63^

### Solid phase binding assay

Human recombinant RANKL (OriGene, Germany) was bound for 2 h under constant agitation to Well-Coated™ Nickel (G-Biosciences) previously washed with PBST. Unbound protein was removed by repeated washing with PBST. RANKL-coated plates were incubated overnight at 4 °C with human recombinant OMD (R&D Systems). The OMD bound to the coated plate was detected using the primary biotinylated polyclonal goat antibody anti-human OMD (R&D Systems, ref: BAF2884, 0.4 μg·mL^−1^). Plates were incubated with streptavidin-POD (Roche, dilution 1:25 000) for 30 min for detection. Finally, plates were read at 450 nm after applying TMB (TMBplus2, D-Tek, Denmark) for 8 min. The direct binding between OMD and RANKL was assessed with a fixed concentration of RANKL (0.2 μg·mL^−1^) and decreasing concentrations of OMD (1 000 to 15.65 ng·L^−1^ by serial 2X dilution), with the negative control missing RANKL; and with decreasing concentrations of RANKL (800 to 6.25 ng·mL^−1^ by serial 2X dilution) and fixed concentration of OMD (0.5 μg·mL^−1^), with the negative control missing OMD.

### Mouse osteoclast culture

WT mice of at least 4 months of age were used to collect bone marrow cells. The bone marrow of the femur and the tibia was flushed with 10 mL of αMEM containing 10% FBS, 100 U per mL penicillin, and 100 mg·mL^−1^ streptomycin. Cells were strained through a 70 µm filter and then centrifuged at 1 200  r·min^−1^ for 7 min at 22 °C. After centrifugation, cells were suspended in 12 mL of media containing 5 ng·mL^−1^ M-CSF in a petri dish and incubated overnight at 37 °C. The nonadherent cells were centrifuged at 1 200 r·min^−1^ for 7 min at 4 °C the next day. The cells were suspended in the osteoclast differentiation medium αMEM containing 10% FBS, 100 U per mL penicillin, 100 mg·mL^−^^1^ streptomycin, 30 ng·mL^−1^ M-CSF and 10 ng·mL^−1^ RANKL. For the treatment conditions, 10 and 40 ng·mL^−1^ recombinant mouse OMD (R&D Systems) were preincubated for at least 15 min with RANKL and M-CSF prior to addition to the suspension of the cells. Cells were seeded (525 000 cells per cm^2^) in 24-well plates. Cells were maintained until 4 days of differentiation and were stained with a TRAP staining kit (Sigma-Aldrich) according to the manufacturer’s instructions.

### Assay in the serum for bone turnover markers

Serum was extracted from the blood of 16-month-old mice collected at euthanasia. The level of P1NP was measured by Rat/Mouse P1NP ELISA (Immunodiagnostic Systems, Boldon, UK), and the level of TRAcP 5b was measured by the Mouse TRAP Assay, a solid phase immunofixed enzyme activity assay (Immunodiagnostic Systems, Boldon, UK), according to the manufacturer’s protocol.

### Mechanical testing

The biomechanical properties of the tibia of 16-month-old mice were determined using a three-point bending test in an Instron 5565 tensile testing machine. Tibiae were stored in PBS, and the remaining soft tissues and fibulae were carefully removed. The samples were assessed at room temperature on a special holding device on their anteroposterior axis. Force was applied on the midpoint of the tibia diaphysis with a 100 N load cell at 8 mm separation (span length) with a perpendicular constant speed of 0.05 mm·s^−1^ and with a preload of 1 N until it fractured. From the load‒deformation curve, the values for the maximal load (N) and stiffness (N·mm^−1^) were obtained.^64^

### Statistical analysis

The results were statistically analyzed using GraphPad Prism 6.0. The tests performed and statistical significance are indicated in the figure legends, with *P* values < 0.05 considered statistically significant.

### Supplementary information

Supplementary Figures

Supplementary Table S1

Supplementary Table S2

## Data Availability

Data generated and analyzed during this study are included in this published article (and its Supplementary Information files). The scripts generated for histomorphometry analysis using Qupath are deposited in GitHub and publicly accessible for cartilage (Toluidine blue) and bone (Safranin-O Fast Green) analysis at 10.5281/zenodo.7898818 and 10.5281/zenodo.7899774, respectively. The transcriptomic data analyzed for this study are deposited in the Gene Expression Omnibus (GEO) repository under accession number GSE230198, which is publicly accessible at https://www.ncbi.nlm.nih.gov/geo/query/acc.cgi?acc=GSE230198.
