# Supplementary material for: Osteomodulin downregulation is associated with osteoarthritis development
Source: Bone Res. 2023 Sep 20;11:49. doi: 10.1038/s41413-023-00286-5 (PMC10511717; doi:10.1038/s41413-023-00286-5)
Supplement: Supplementary file 1 — Supplementary Figures [file 41413_2023_286_MOESM1_ESM.docx]

**Supplementary Figure S1**


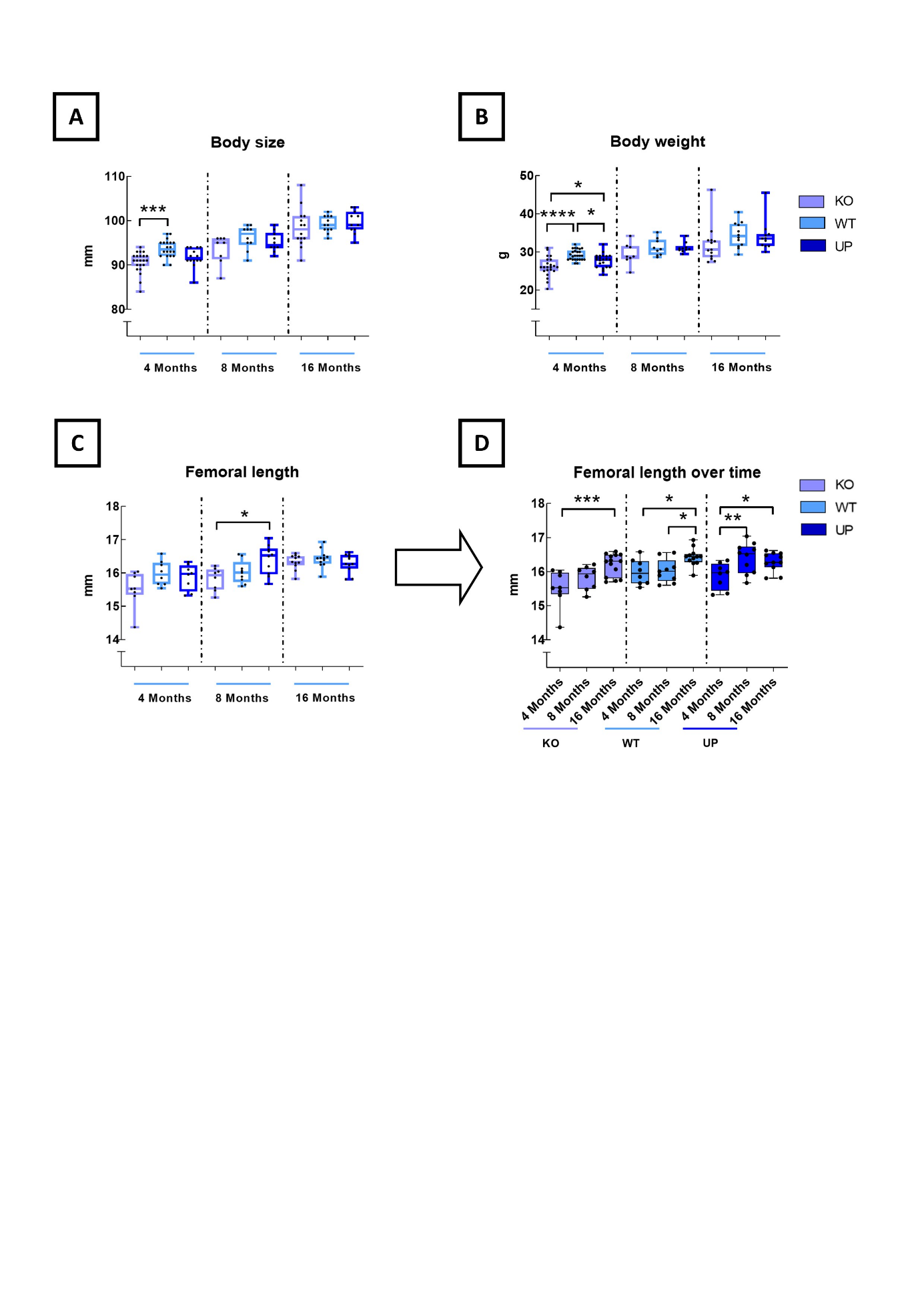


Supplementary Figure S1:Macro observations of the cartilage of the knee joint of male mice. Mice that were candidates for the DMM surgery were added to the macro observations when possible. The macro observations were conducted at 4, 8 and 16 months. (A) For the body size: n=22 for the KO, n=20 for the WT and n=14 for the UP at 4 months; n=8 for the KO and n=10 for the WT and UP at 8 months; n=14 for the KO, n=12 for the WT and n=11 for the UP at 16 months. (B) For the body weight: n=22 for the KO, n=26 for the WT and n=22 for the UP at 4 months; n=8 for the KO and n=9 for the WT and n=10 for the UP at 8 months; n=13 for the KO, n=12 for the WT and n=11 for the UP at 16 months. (C) For the femoral length: n=8 for each genotype at 4 months; n=8 for the KO and n=9 for the WT and n=10 for the UP at 8 months; n=11 for the KO, n=12 for the WT and n=11 for the UP at 16 months. (D) The femoral length was displayed to show the evolution of the femurs over time within the genotypes. (A-C) Ordinary one-way ANOVA was performed to compare the body size, the body weight, and the femoral length between the genotypes, at each time-point. Kruskal-Wallis was performed when the distribution was not Gaussian. (D) Two-way ANOVA was performed to examine the influence of time on the femoral length inside each genotype. The data were plotted as a box plot showing all points with differences being considered significant at p-values<0.05 (*p<0.05, **p≤0.01, ***p≤0.001, ****p≤0.0001).

**Supplementary Figure S2**


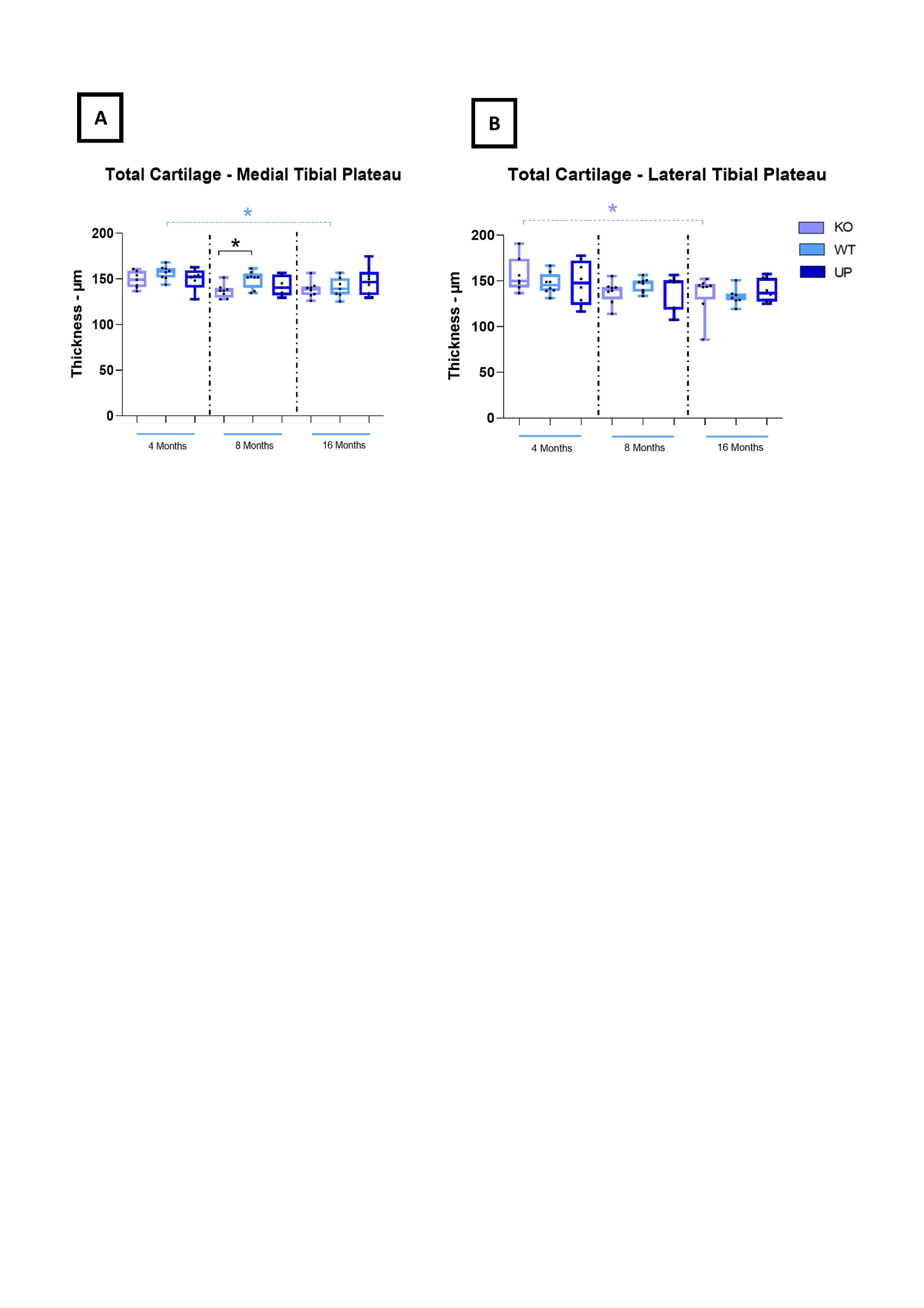


Supplementary Figure S2: Histomorphometry of the cartilage was performed with QuPath at 4, 8, and 16 months. Knee joints of male mice were stained with Toluidine Blue and the thickness of the total cartilage (including non-calcified and calcified cartilage) was measured. (A) For the medial tibial plateau: n=7 for the KO, n=8 for the WT and UP at 4 months; n=8 for the KO and WT and n=7 for the UP at 8 months; n=8 for the KO, n=7 for the WT and n=9 for the UP at 16 months. (B) For the lateral tibial plateau: n=7 for the KO, n=8 for the WT and UP at 4 months; n=8 for all the genotypes at 8 months; n=8 for the KO, n=7 for the WT and n=9 for the UP at 16 months. Two-way ANOVA was performed to evaluate the genotype effect (in black) and the time effect inside a genotype (in the corresponding color). The data were plotted as a box plot showing all points with differences being considered significant at p-values<0.05 (*p<0.05).

**Supplementary Figure S3**


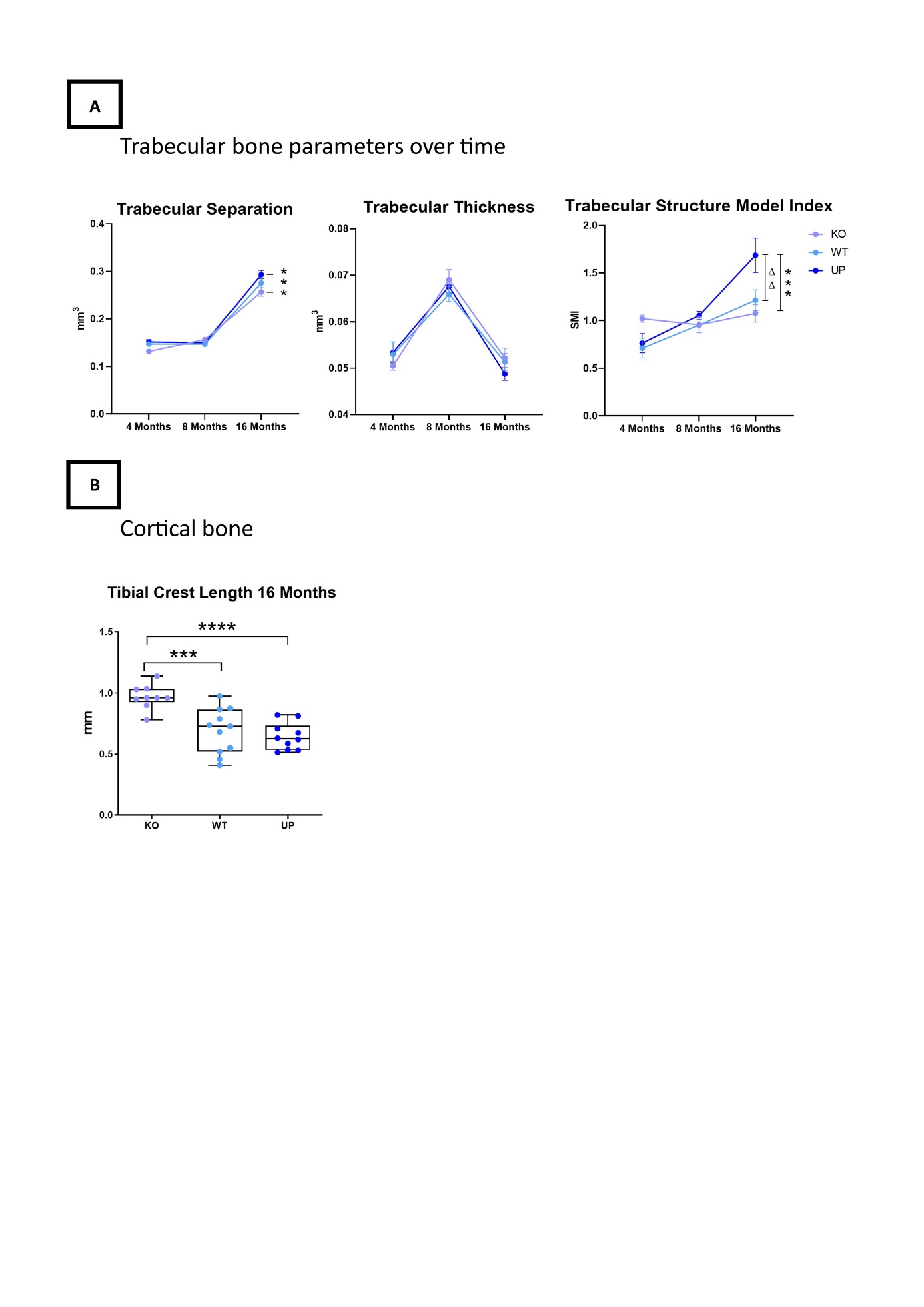


Supplementary Figure S3: µCT analysis on the metaphysis of the tibia of the male mice at 4, 8 and 16 months (A) The trabecular bone parameters over time, with the trabecular separation, the trabecular thickness and the trabecular structure model index. Two-Way ANOVA was performed with error bars representing ± SEM and differences being considered significant at p-values<0.05, * represents significant differences between the KO and the UP and Δ represents significant differences between the WT and the UP (*/Δp<0.05, **/ΔΔp≤0.01, ***p≤0.001). (B) The length of the tibial crest of 16-month-old mice. One-Way ANOVA was performed, and the data were plotted as a box plot showing all points with differences being considered significant at p-values<0.05 (*p<0.05, **p≤0.01, ***p≤0.001, ****p≤0.0001). At 4 months: n=8 for each genotype; at 8 months: n=8 for the KO, n=10 for the WT and UP; at 16 months: n=9 for the KO, n=11 for the WT, and n=10 for the UP.

**Supplementary Figure S4**


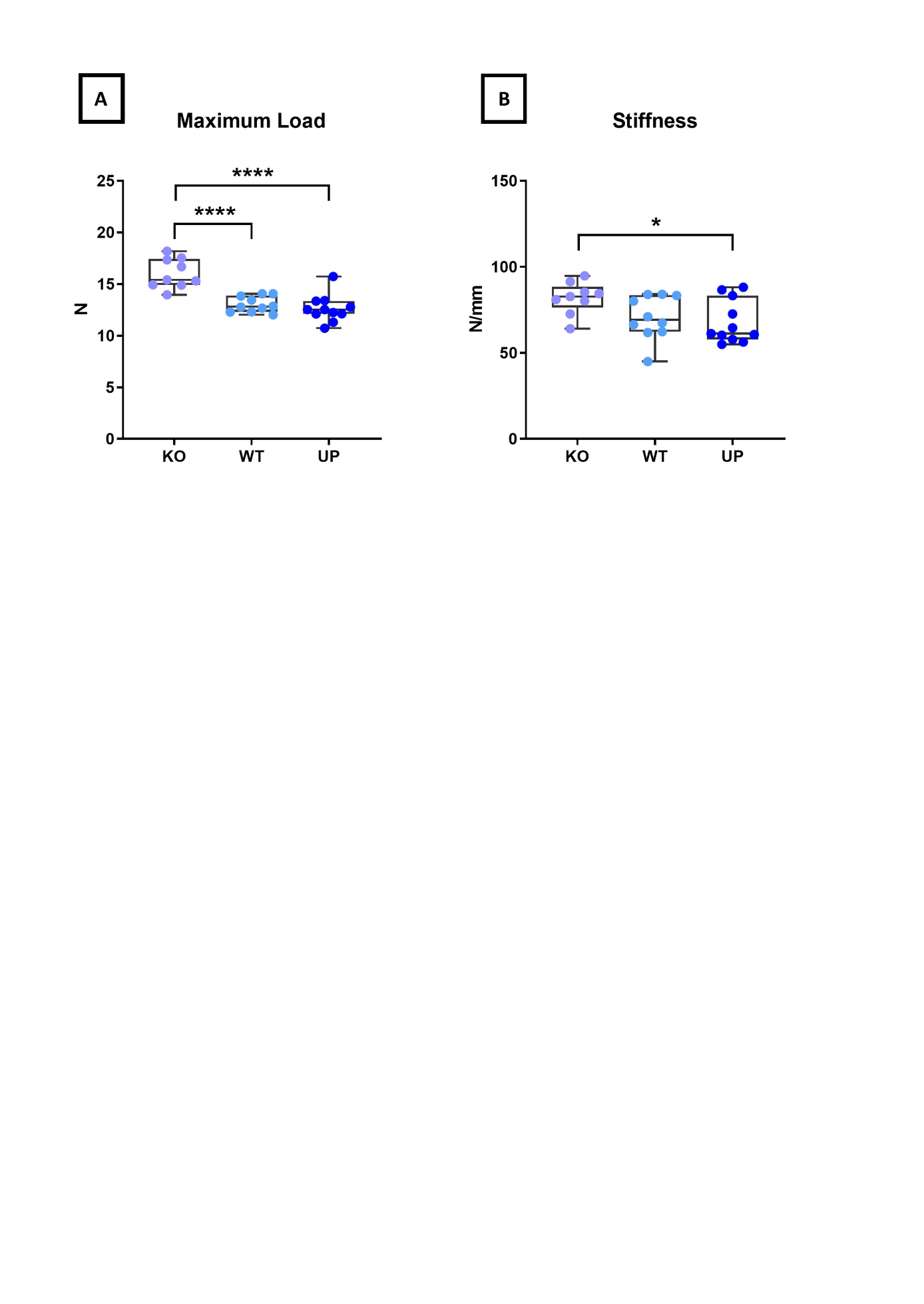


Supplementary Figure S4: Biomechanical testing of the tibia from 16 months mice. (A) The maximum load (N) and (B) bone’s stiffness (N/mm) were assessed using a three-point bending test with n=9 for the KO, n=10 for the WT and n=11 for the UP. One-Way ANOVA was performed, the data were plotted as a box plot showing all points with differences being considered significant at p-values<0.05 (*p<0.05, ****p≤0.0001).

**Supplementary Figure S5**


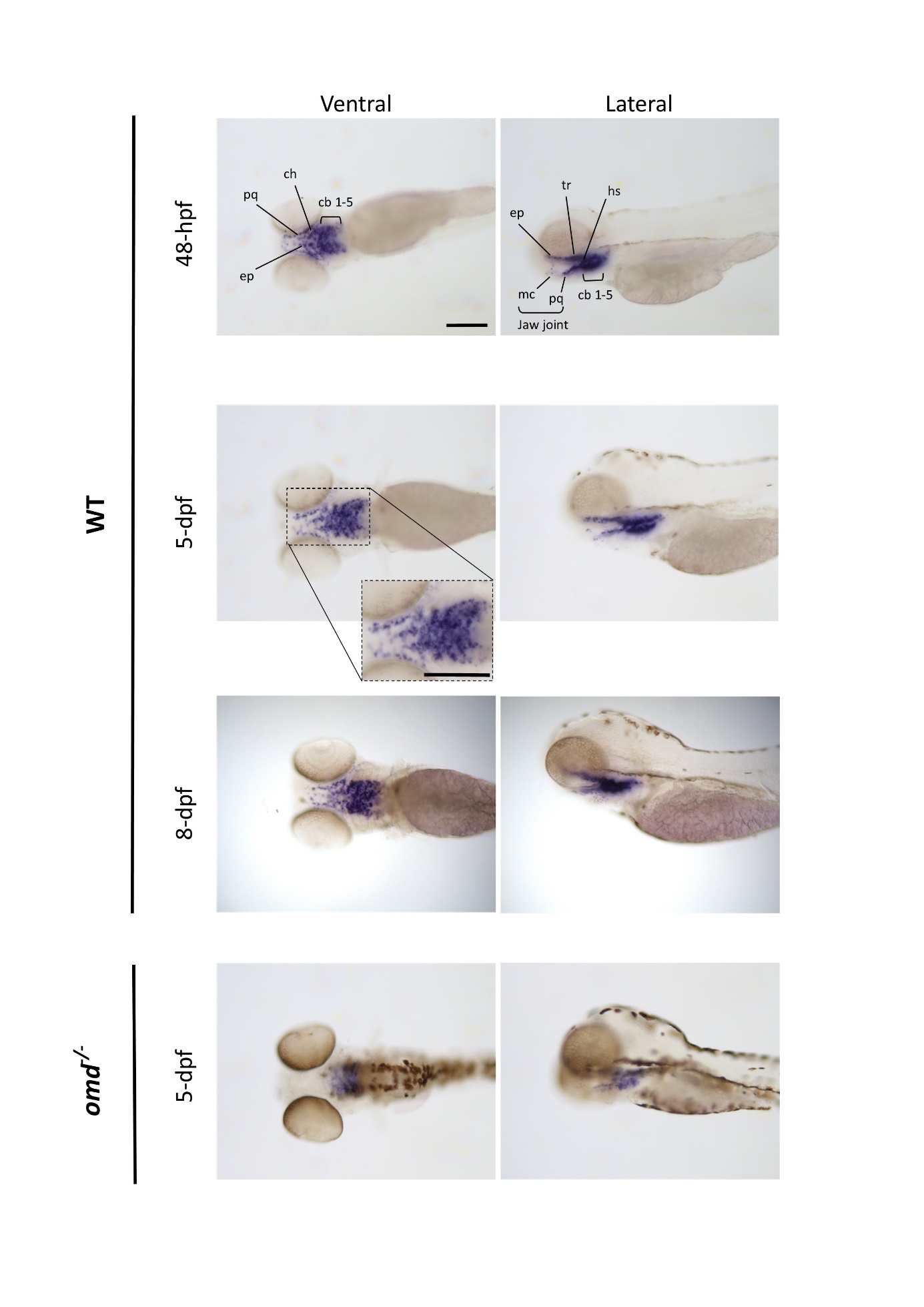


Supplementary Figure S5: Whole mount in situ hybridization for the omd mRNA on WT zebrafish larvae at 48-hpf, 5-dpf and 8-dpf and on omd^-/-^ larvae at 5-dpf. Ventral and lateral views are illustrated. Scale bar=200 µm. Cartilage structures are annotated as followed Ep: Ethmoid plate; Pq: Palatoquadrate; Ch: Ceratohyal; Cb: Ceratobranchial arches; Mc: Meckel’s Cartilage; Tr: trabecula; Hs: Hyosymplectic cartilage. The meckels’s cartilage and the palatoquadrate articulate themselves into the jaw joint.

**Supplementary Figure S6**


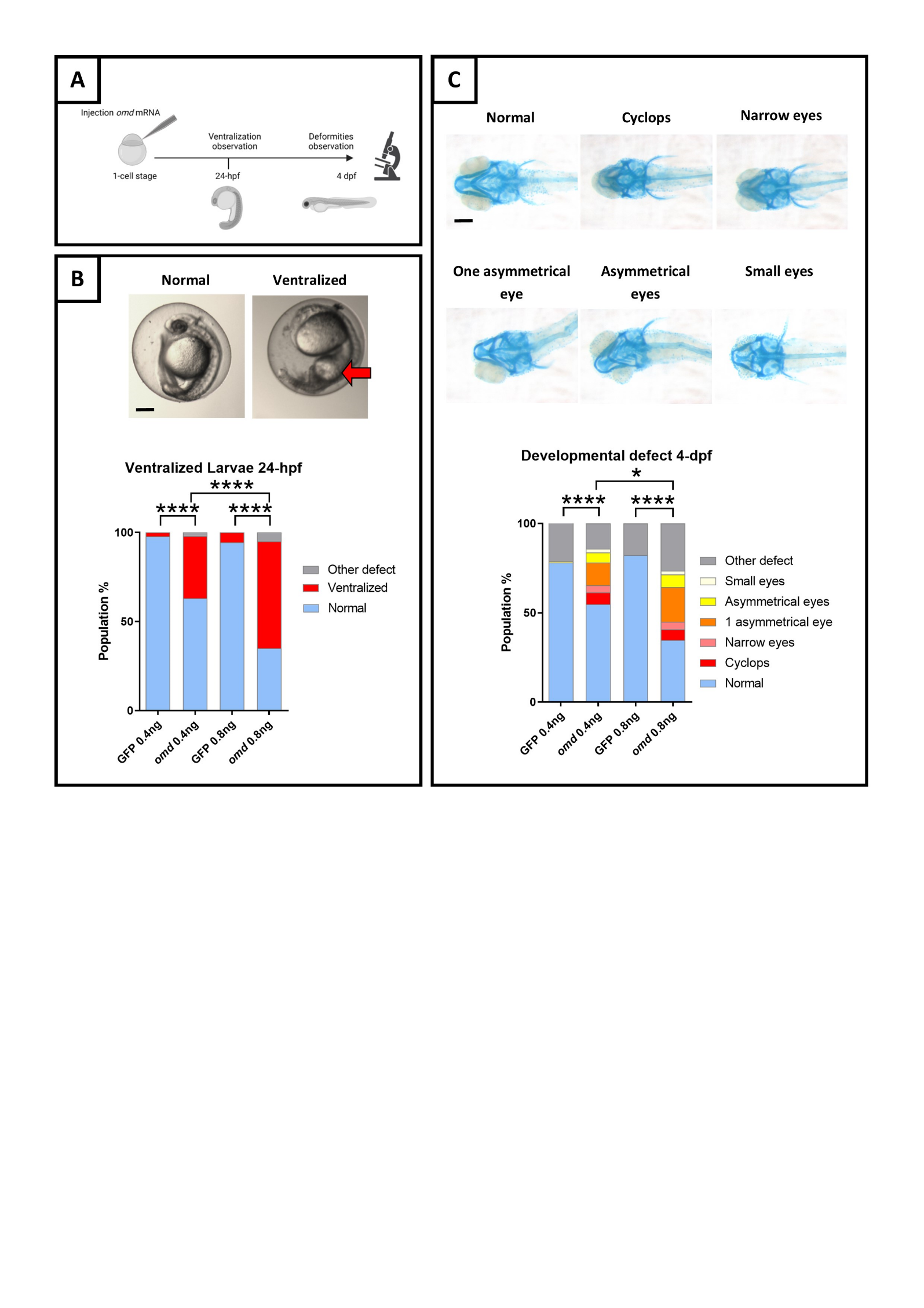


Supplementary Figure S6: Analysis of the overexpression of omd in the zebrafish. (A) The overexpression model was generated by the injection of the zebrafish mRNA of omd in the 1-cell zygote followed by the observation of the ventralization at 24-hpf and the developmental deformities at 4-dpf. (B) The percentage of ventralized larvae was reported at 24-hpf after the injection of 0.4 ng (n=314) or 0.8 ng (n=103) of omd. The injection of 0.4 ng (n=301) or 0.8 ng (n=72) of the mRNA of the GFP served as a control. The data were plotted as a percentage of the observed class in stacked columns. A Chi-square test was performed with differences being considered significant at p-values<0.05 (****p≤0.0001). A normal embryo and ventralized embryo with a red arrow pointing to the ventralization are illustrated. Scale bar=200 µm. (C) The percentage of developmental deformities presented by the larvae was reported at 4-dpf after the injection of 0.4 ng (n=367) or 0.8 ng (n=96) of omd. The injection of 0.4 ng (n=169) or 0.8 ng (n=79) of the mRNA of the GFP served as a control. A different class of deformities was attributed: normal, cyclops, narrow eyes, one asymmetrical eye, asymmetrical eyes, small eyes and other defect which included non-specific defects. The larvae were stained with Alcian Blue and the representative illustration of each class was selected. Scale bar=200 µm. The data were plotted as a percentage of the observed class in stacked columns. A Chi-square test was performed with differences being considered significant at p-values < 0.05 (*p<0.05, ****p≤0.0001).

**Supplementary Figure S7**


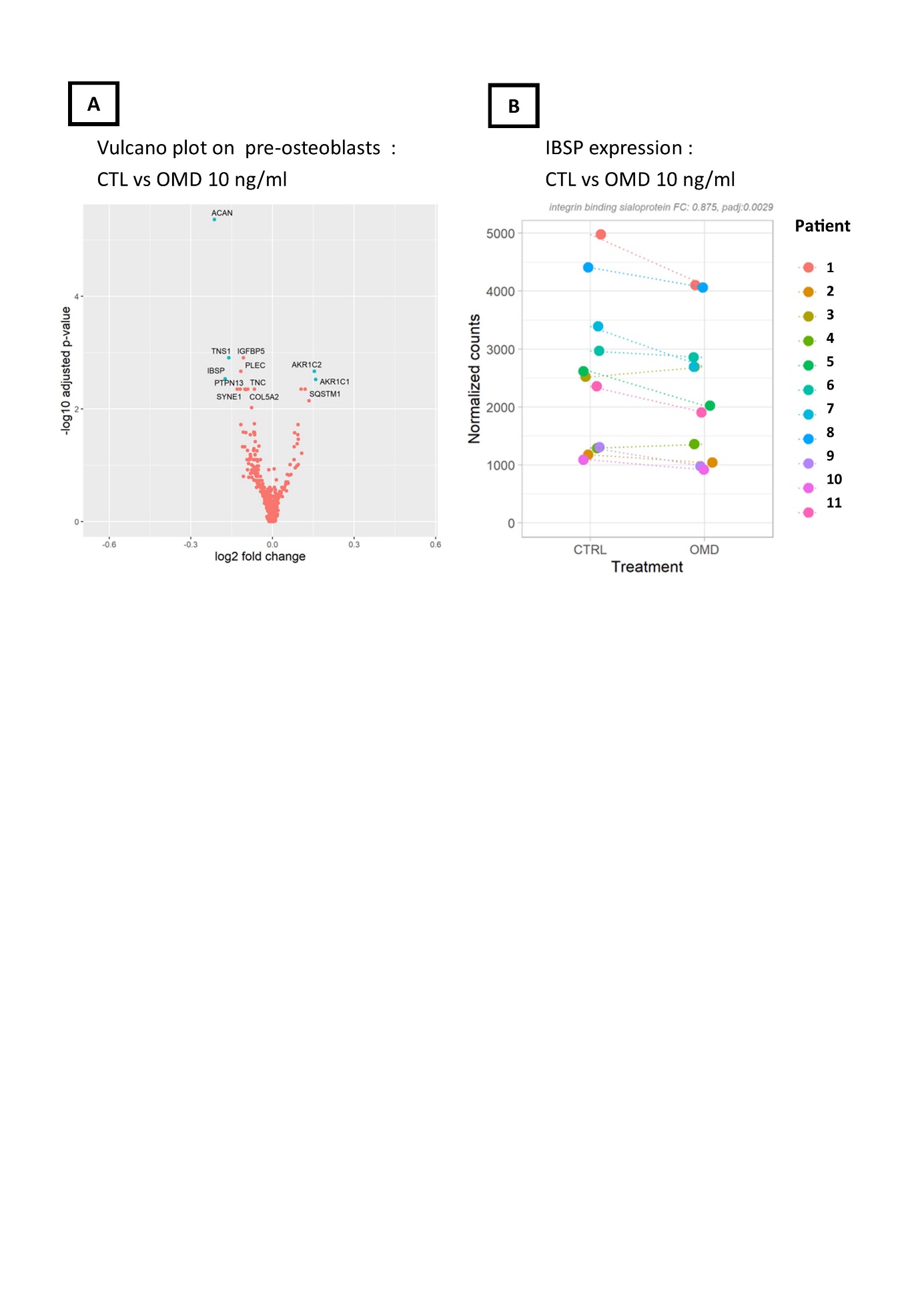


Supplementary Figure S7: RNA-seq analysis of primary human pre-osteoblast cultures treated with 10 ng/ml of rhOMD during 72-h with n=11 (6 men and 5 women). (A) Vulcano plot with Threshold padj < 0.01 and Log2FoldChange. (B) IBSP gene expression comparison.

**Supplementary Figure S8**


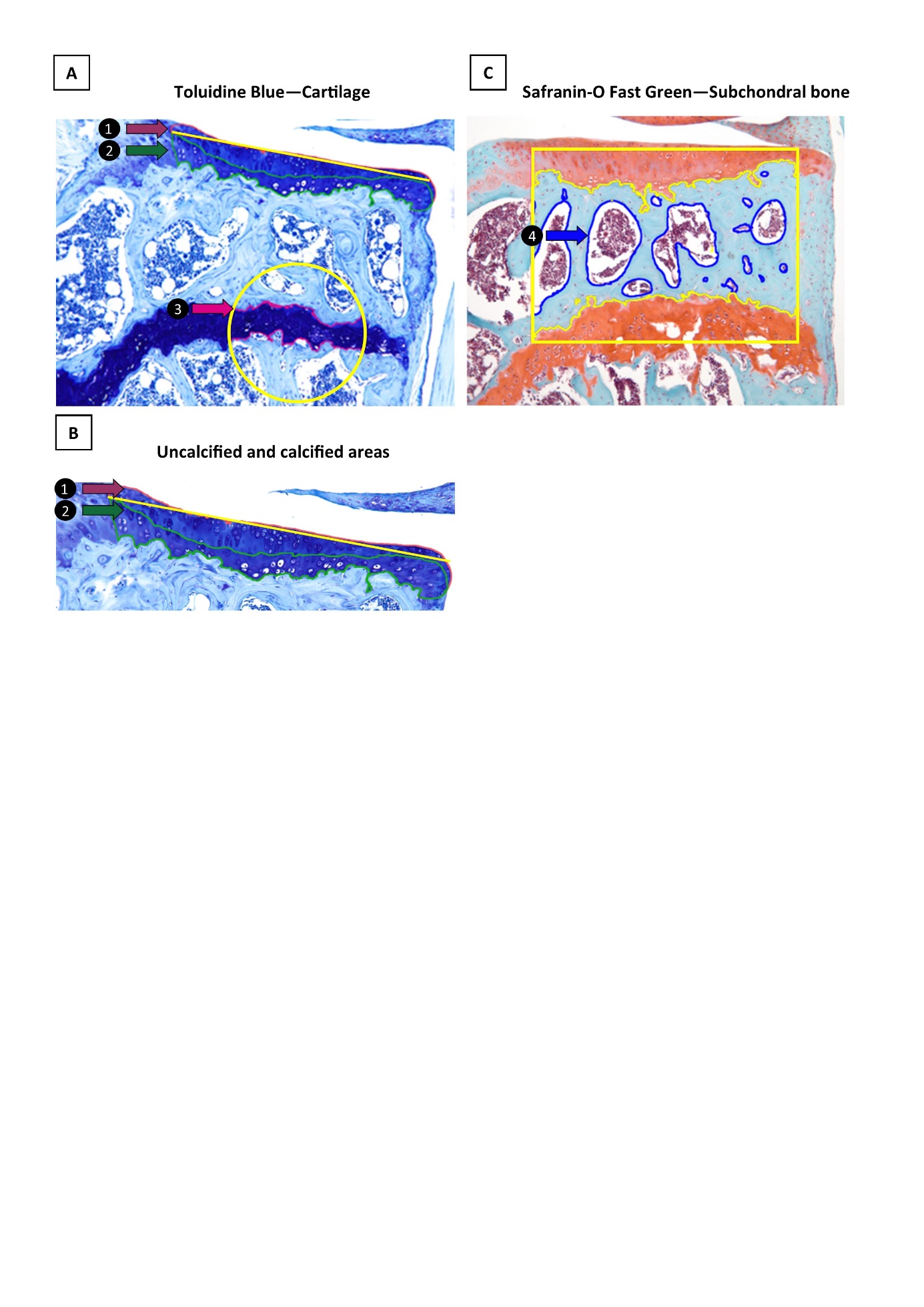


Supplementary Figure S8: Illustration of the ROI measured with QuPath for the histomorphometry analysis. (A, B) Toluidine Blue staining was used for uncalcified (1) and calcified (2) cartilage measurements. The yellow line was drawn to measure the plate length. Inside the yellow circle, the area of the growth plate was defined (3). (C) The Safranin-O Fast Green was used to measure the subchondral bone volume. The subchondral bone volume was measured inside the yellow box and calculated using the formula TV – (Bone marrow volume (4) + cartilage volume).
