# Supplementary material for: Osteomodulin downregulation is associated with osteoarthritis development
Source: Bone Res. 2023 Sep 20;11:49. doi: 10.1038/s41413-023-00286-5 (PMC10511717; doi:10.1038/s41413-023-00286-5)
Supplement: Supplementary file 2 — Supplementary Table S1 [file 41413_2023_286_MOESM2_ESM.docx]

**Supplementary Table S1**

Supplementary Table S1: Analysis of the gait development from the CatWalkXT of 4, 8 and 16-month-old male mice. Parameters of the front paws and the hind paws are reported. The first column corresponds to the gait parameter with its specific unit; the second column corresponds to the genotype, the third column corresponds to the reported value, the fourth, fifth and sixth columns are the reported p-values between KO and WT, KO and UP, WT and UP respectively. At 4 months: n=8 for each genotype; at 8 months n=8 for each genotype; at 16 months: n=14 for the KO, n=12 for the WT and n=10 for the UP. One-Way ANOVA was performed when the distribution was Gaussian and Kruskal-Wallis was performed when the distribution was not Gaussian with differences being considered significant at p-values<0.05 (*p<0.05, **p≤0.01, ***p≤0.001).

**4 months: Front Paws**

| Parameter | Genotype | Value | KO vs WT | KO vs UP | UP vs WT |
| --- | --- | --- | --- | --- | --- |

| Stand Index | KO | -3,135 | 0,6056 | 0,9594 | 0,7708 |
| --- | --- | --- | --- | --- | --- |
|  | WT | -2,702 |  |  |  |
|  | UP | -3,012 |  |  |  |
| Stand  (s) | KO | 0,1982 | 0,9999 | 0,9999 | 0,9999 |
|  | WT | 0,1968 |  |  |  |
|  | UP | 0,1825 |  |  |  |
| Max Contact Area  (cm^2^) | KO | 1,172 | 0,4027 | **0,0033 (**)** | 0,0610 |
|  | WT | 1,073 |  |  |  |
|  | UP | 0,8901 |  |  |  |
| Print Area  (cm^2^) | KO | 1,364 | 0,3367 | **0,0032 (**)** | 0,0762 |
|  | WT | 1,233 |  |  |  |
|  | UP | 1,024 |  |  |  |
| Swing  (s) | KO | 0,1191 | **0,0400 (*)** | **0,0362 (*)** | 0,9999 |
|  | WT | 0,1475 |  |  |  |
|  | UP | 0,1427 |  |  |  |
| Swing Speed  (cm) | KO | 75,67 | 0,5725 | 0,5373 | 0,9999 |
|  | WT | 66,77 |  |  |  |
|  | UP | 67,95 |  |  |  |
| Stride Length  (cm) | KO | 8,430 | 0,2212 | 0,1306 | 0,9499 |
|  | WT | 9,074 |  |  |  |
|  | UP | 9,188 |  |  |  |
| Step Cycle  (s) | KO | 0,3153 | 0,7737 | 0,9999 | 0,9999 |
|  | WT | 0,3429 |  |  |  |
|  | UP | 0,3206 |  |  |  |
| Single Stance  (s) | KO | 0,1191 | **0,0296 (*)** | **0,0362 (*)** | 0,9999 |
|  | WT | 0,1443 |  |  |  |
|  | UP | 0,1412 |  |  |  |
| Initial dual stance  (s) | KO | 0,03823 | 0,0935 | **0,0021 (**)** | 0,2172 |
|  | WT | 0,02829 |  |  |  |
|  | UP | 0,02049 |  |  |  |
| Terminal dual stance  (s) | KO | 0,03836 | **0,0470 (*)** | **0,0047 (**)** | 0,5603 |
|  | WT | 0,02713 |  |  |  |
|  | UP | 0,02255 |  |  |  |
| Base of support  (cm) | KO | 2,106 | **0,0140 (*)** | 0,4637 | 0,1610 |
|  | WT | 1,688 |  |  |  |
|  | UP | 1,944 |  |  |  |
| Intensity | KO | 0,08859 | 0,5010 | 0,0521 | 0,1401 |
|  | WT | 0,09884 |  |  |  |
|  | UP | 0,1272 |  |  |  |

**4 months: Hind Paws**

| Parameter | Genotype | Value | KO vs WT | KO vs UP | UP vs WT |
| --- | --- | --- | --- | --- | --- |

| Stand Index | KO | -10,62 | **0,0061 (**)** | **0,0153 (*)** | 0,9136 |
| --- | --- | --- | --- | --- | --- |
|  | WT | -7,544 |  |  |  |
|  | UP | 7,903 |  |  |  |
| Stand  (s) | KO | 0,1850 | 0,0931 | 0,5373 | 0,9999 |
|  | WT | 0,2091 |  |  |  |
|  | UP | 0,1975 |  |  |  |
| Max Contact Area  (cm^2^) | KO | 0,7649 | **0,0134 (*)** | 0,1267 | 0,5320 |
|  | WT | 1,074 |  |  |  |
|  | UP | 0,9663 |  |  |  |
| Print Area  (cm^2^) | KO | 0,9344 | **0,0142 (*)** | 0,2787 | 0,2975 |
|  | WT | 1,292 |  |  |  |
|  | UP | 1,116 |  |  |  |
| Swing  (s) | KO | 0,1319 | 0,6208 | 0,7914 | 0,9560 |
|  | WT | 0,1226 |  |  |  |
|  | UP | 0,1254 |  |  |  |
| Swing Speed  (cm/s) | KO | 67,92 | 0,4127 | 0,5038 | 0,9999 |
|  | WT | 77,41 |  |  |  |
|  | UP | 74,37 |  |  |  |
| Stride Length  (cm) | KO | 8,330 | 0,4284 | 0,0898 | 0,6104 |
|  | WT | 8,783 |  |  |  |
|  | UP | 9,126 |  |  |  |
| Step Cycle  (s) | KO | 0,3108 | 0,7731 | 0,8602 | 0,9855 |
|  | WT | 0,3285 |  |  |  |
|  | UP | 0,3243 |  |  |  |
| Single Stance  (s) | KO | 0,1191 | 0,9914 | 0,7499 | 0,8200 |
|  | WT | 0,1200 |  |  |  |
|  | UP | 0,1242 |  |  |  |
| Initial dual stance  (s) | KO | 0,03142 | 0,0535 | 0,3594 | 0,9999 |
|  | WT | 0,04169 |  |  |  |
|  | UP | 0,03783 |  |  |  |
| Terminal dual stance  (s) | KO | 0,03356 | 0,0778 | 0,5038 | 0,9999 |
|  | WT | 0,04485 |  |  |  |
|  | UP | 0,03976 |  |  |  |
| Base of support  (cm) | KO | 3,516 | 0,8857 | 0,8160 | 0,9893 |
|  | WT | 3,609 |  |  |  |
|  | UP | 3,637 |  |  |  |
| Intensity | KO | 0,1530 | 0,1829 | 0,9999 | 0,8192 |
|  | WT | 0,09976 |  |  |  |
|  | UP | 0,1218 |  |  |  |

**8 months: Front Paws**

| Parameter | Genotype | Value | KO vs WT | KO vs UP | UP vs WT |
| --- | --- | --- | --- | --- | --- |

| Stand Index | KO | -2,976 | 0,4716 | 0,9999 | 0,9999 |
| --- | --- | --- | --- | --- | --- |
|  | WT | -2,397 |  |  |  |
|  | UP | -2,732 |  |  |  |
| Stand  (s) | KO | 0,2172 | 0,5961 | 0,9099 | 0,3607 |
|  | WT | 0,2356 |  |  |  |
|  | UP | 0,2095 |  |  |  |
| Max Contact Area  (cm^2^) | KO | 1,030 | **0,0120 (*)** | 0,7729 | 0,0529 |
|  | WT | 1,256 |  |  |  |
|  | UP | 1,079 |  |  |  |
| Print Area  (cm^2^) | KO | 1,178 | **0,0140 (*)** | 0,6791 | 0,0832 |
|  | WT | 1,451 |  |  |  |
|  | UP | 1,252 |  |  |  |
| Swing  (s) | KO | 0,1229 | 0,1553 | **0,0486 (*)** | 0,9999 |
|  | WT | 0,1500 |  |  |  |
|  | UP | 0,1533 |  |  |  |
| Swing Speed  (cm/s) | KO | 68,47 | 0,7632 | 0,7452 | 0,9995 |
|  | WT | 64,77 |  |  |  |
|  | UP | 64,60 |  |  |  |
| Stride Length  (cm) | KO | 7,929 | 0,1938 | **0,0336 (*)** | 0,6403 |
|  | WT | 8,892 |  |  |  |
|  | UP | 9,379 |  |  |  |
| Step Cycle  (s) | KO | 0,3408 | 0,1686 | 0,6090 | 0,9999 |
|  | WT | 0,3842 |  |  |  |
|  | UP | 0,3604 |  |  |  |
| Single Stance  (s) | KO | 0,1228 | 0,1430 | 0,0535 | 0,9999 |
|  | WT | 0,1481 |  |  |  |
|  | UP | 0,1516 |  |  |  |
| Initial dual stance  (s) | KO | 0,04792 | 0,8489 | 0,0759 | 0,2054 |
|  | WT | 0,04300 |  |  |  |
|  | UP | 0,02712 |  |  |  |
| Terminal dual stance  (s) | KO | 0,04756 | 0,9554 | 0,0559 | 0,0986 |
|  | WT | 0,04531 |  |  |  |
|  | UP | 0,02831 |  |  |  |
| Base of support  (cm) | KO | 2,002 | 0,5068 | 0,5391 | 0,9984 |
|  | WT | 1,864 |  |  |  |
|  | UP | 1,870 |  |  |  |
| Intensity | KO | 0,1131 | 0,9999 | 0,6093 | 0,9999 |
|  | WT | 0,09739 |  |  |  |
|  | UP | 0,09320 |  |  |  |

**8 months: Hind Paws**

| Parameter | Genotype | Value | KO vs WT | KO vs UP | UP vs WT |
| --- | --- | --- | --- | --- | --- |

| Stand Index | KO | -8,854 | 0,2133 | 0,1638 | 0,9867 |
| --- | --- | --- | --- | --- | --- |
|  | WT | -6,898 |  |  |  |
|  | UP | -6,723 |  |  |  |
| Stand  (s) | KO | 0,2285 | 0,6897 | 0,8393 | 0,3614 |
|  | WT | 0,2456 |  |  |  |
|  | UP | 0,2168 |  |  |  |
| Max Contact Area  (cm^2^) | KO | 0,7735 | **0,0002 (***)** | **0,0150 (*)** | 0,1838 |
|  | WT | 1,281 |  |  |  |
|  | UP | 1,092 |  |  |  |
| Print Area  (cm^2^) | KO | 0,9831 | **0,0002 (***)** | **0,0230 (*)** | 0,1125 |
|  | WT | 1,519 |  |  |  |
|  | UP | 1,293 |  |  |  |
| Swing  (s) | KO | 0,1061 | **0,0105 (*)** | **0,0345 (*)** | 0,8521 |
|  | WT | 0,1339 |  |  |  |
|  | UP | 0,1293 |  |  |  |
| Swing Speed  (cm/s) | KO | 75,28 | 0,6475 | 0,9999 | 0,9999 |
|  | WT | 67,74 |  |  |  |
|  | UP | 73,17 |  |  |  |
| Stride Length  (cm) | KO | 7,764 | 0,2493 | **0,0447 (*)** | 0,6284 |
|  | WT | 8,665 |  |  |  |
|  | UP | 9,174 |  |  |  |
| Step Cycle  (s) | KO | 0,2879 | 0,2139 | 0,9999 | 0,8662 |
|  | WT | 0,3027 |  |  |  |
|  | UP | 0,2862 |  |  |  |
| Single Stance  (s) | KO | 0,1050 | **0,0104 (*)** | **0,0390 (*)** | 0,8197 |
|  | WT | 0,1320 |  |  |  |
|  | UP | 0,1270 |  |  |  |
| Initial dual stance  (s) | KO | 0,06246 | 0,7260 | 0,2462 | 0,6554 |
|  | WT | 0,05460 |  |  |  |
|  | UP | 0,04554 |  |  |  |
| Terminal dual stance  (s) | KO | 0,06126 | 0,8707 | 0,2389 | 0,4838 |
|  | WT | 0,05661 |  |  |  |
|  | UP | 0,04579 |  |  |  |
| Base of support  (cm) | KO | 3,917 | 0,7297 | 0,9999 | 0,9999 |
|  | WT | 4,024 |  |  |  |
|  | UP | 4,007 |  |  |  |
| Intensity | KO | 0,1461 | **0,0016 (**)** | **0,0027 (**)** | 0,9722 |
|  | WT | 0,09121 |  |  |  |
|  | UP | 0,09427 |  |  |  |

**16 months: Front Paws**

| Parameter | Genotype | Value | KO vs WT | KO vs UP | UP vs WT |
| --- | --- | --- | --- | --- | --- |

| Stand Index | KO | -3,510 | 0,6897 | **0,0414 (*)** | 0,6034 |
| --- | --- | --- | --- | --- | --- |
|  | WT | -2,574 |  |  |  |
|  | UP | -2,114 |  |  |  |
| Stand  (s) | KO | 0,2083 | 0,6504 | 0,2633 | 0,7525 |
|  | WT | 0,2245 |  |  |  |
|  | UP | 0,2388 |  |  |  |
| Max Contact Area  (cm^2^) | KO | 0,9117 | 0,1656 | 0,0987 | 0,9345 |
|  | WT | 1,022 |  |  |  |
|  | UP | 1,045 |  |  |  |
| Print Area  (cm^2^) | KO | 1,037 | 0,1390 | 0,1265 | 0,9889 |
|  | WT | 1,166 |  |  |  |
|  | UP | 1,177 |  |  |  |
| Swing  (s) | KO | 0,1127 | **0,0110 (*)** | **0,0005 (***)** | 0,4263 |
|  | WT | 0,1430 |  |  |  |
|  | UP | 0,1565 |  |  |  |
| Swing Speed  (cm/s) | KO | 80,19 | 0,1677 | **0,0057 (**)** | 0,2918 |
|  | WT | 69,05 |  |  |  |
|  | UP | 59,08 |  |  |  |
| Stride Length  (cm) | KO | 8,130 | **0,0114 (*)** | 0,4293 | 0,2553 |
|  | WT | 9,176 |  |  |  |
|  | UP | 8,580 |  |  |  |
| Step Cycle  (s) | KO | 0,3204 | 0,1834 | **0,0251 (*)** | 0,5799 |
|  | WT | 0,3673 |  |  |  |
|  | UP | 0,3957 |  |  |  |
| Single Stance  (s) | KO | 0,1102 | **0,0255 (*)** | **0,0008 (***)** | 0,7841 |
|  | WT | 0,1422 |  |  |  |
|  | UP | 0,1545 |  |  |  |
| Initial dual stance  (s) | KO | 0,04890 | 0,2393 | 0,6400 | 0,9999 |
|  | WT | 0,03957 |  |  |  |
|  | UP | 0,04172 |  |  |  |
| Terminal dual stance  (s) | KO | 0,04986 | 0,1950 | 0,4125 | 0,9226 |
|  | WT | 0,03952 |  |  |  |
|  | UP | 0,04196 |  |  |  |
| Base of support  (cm) | KO | 2,189 | 0,4017 | **0,0357 (*)** | 0,3963 |
|  | WT | 2,040 |  |  |  |
|  | UP | 1,875 |  |  |  |
| Intensity | KO | 0,1370 | 0,2138 | 0,5193 | 0,9999 |
|  | WT | 0,1060 |  |  |  |
|  | UP | 0,1091 |  |  |  |

**16 months: Hind Paws**

| Parameter | Genotype | Value | KO vs WT | KO vs UP | UP vs WT |
| --- | --- | --- | --- | --- | --- |

| Stand Index | KO | -7,240 | 0,9999 | 0,9999 | 0,9999 |
| --- | --- | --- | --- | --- | --- |
|  | WT | -6,534 |  |  |  |
|  | UP | -6,784 |  |  |  |
| Stand  (s) | KO | 0,2204 | 0,5801 | 0,0985 | 0,4962 |
|  | WT | 0,2418 |  |  |  |
|  | UP | 0,2683 |  |  |  |
| Max Contact Area  (cm^2^) | KO | 0,7817 | 0,0566 | 0,1145 | 0,9754 |
|  | WT | 1,007 |  |  |  |
|  | UP | 0,9853 |  |  |  |
| Print Area  (cm^2^) | KO | 0,9933 | 0,1083 | 0,2277 | 0,9543 |
|  | WT | 1,221 |  |  |  |
|  | UP | 1,186 |  |  |  |
| Swing  (s) | KO | 0,09631 | **0,0040 (**)** | **0,0029 (**)** | 0,9565 |
|  | WT | 0,1255 |  |  |  |
|  | UP | 0,1281 |  |  |  |
| Swing Speed  (cm/s) | KO | 87,05 | 0,1386 | **0,0180 (*)** | 0,5844 |
|  | WT | 76,67 |  |  |  |
|  | UP | 70,93 |  |  |  |
| Stride Length  (cm) | KO | 7,936 | **0,0091 (**)** | 0,0951 | 0,6900 |
|  | WT | 9,013 |  |  |  |
|  | UP | 8,707 |  |  |  |
| Step Cycle  (s) | KO | 0,3133 | 0,1067 | **0,0051 (**)** | 0,3767 |
|  | WT | 0,3637 |  |  |  |
|  | UP | 0,3991 |  |  |  |
| Single Stance  (s) | KO | 0,09559 | **0,0055 (**)** | **0,0028 (**)** | 0,9146 |
|  | WT | 0,1236 |  |  |  |
|  | UP | 0,1273 |  |  |  |
| Initial dual stance  (s) | KO | 0,06292 | 0,8214 | 0,9999 | 0,3389 |
|  | WT | 0,05523 |  |  |  |
|  | UP | 0,06931 |  |  |  |
| Terminal dual stance  (s) | KO | 0,06289 | 0,8660 | 0,7988 | 0,5267 |
|  | WT | 0,05807 |  |  |  |
|  | UP | 0,06924 |  |  |  |
| Base of support  (cm) | KO | 3,916 | 0,9984 | 0,7295 | 0,7733 |
|  | WT | 3,908 |  |  |  |
|  | UP | 3,796 |  |  |  |
| Intensity | KO | 0,1646 | **0,0418 (*)** | 0,1269 | 0,9999 |
|  | WT | 0,09608 |  |  |  |
|  | UP | 0,09931 |  |  |  |
